# Deep Coastal Marine Taphonomy: Investigation into Carcass Decomposition in the Saanich Inlet, British Columbia Using a Baited Camera

**DOI:** 10.1371/journal.pone.0110710

**Published:** 2014-10-20

**Authors:** Gail S. Anderson, Lynne S. Bell

**Affiliations:** Centre for Forensic Research, School of Criminology, Simon Fraser University, Burnaby, British Columbia, Canada; Université du Québec à Rimouski, Canada

## Abstract

Decomposition and faunal colonization of a carcass in the terrestrial environment has been well studied, but knowledge of decomposition in the marine environment is based almost entirely on anecdotal reports. Three pig carcasses were deployed in Saanich Inlet, BC, over 3 years utilizing Ocean Network Canada’s VENUS observatory. Each carcass was deployed in late summer/early fall at 99 m under a remotely controlled camera and observed several times a day. Dissolved oxygen, temperature, salinity, density and pressure were continuously measured. Carcass 1 was immediately colonized by *Munida quadrispina*, *Pandalus platyceros* and *Metacarcinus magister,* rapidly scavenged then dragged from view by Day 22. Artifacts specific to each of the crustaceans’ feeding patterns were observed. Carcass 2 was scavenged in a similar fashion. Exposed tissue became covered by *Orchomenella obtusa* (Family Lysianassidae) which removed all the internal tissues rapidly. Carcass 3 attracted only a few *M. quadrispina,* remaining intact, developing a thick filamentous sulphur bacterial mat, until Day 92, when it was skeletonized by crustacea. The major difference between the deployments was dissolved oxygen levels. The first two carcasses were placed when oxygen levels were tolerable, becoming more anoxic. This allowed larger crustacea to feed. However, Carcass 3 was deployed when the water was already extremely anoxic, which prevented larger crustacea from accessing the carcass. The smaller *M. quadrispina* were unable to break the skin alone. The larger crustacea returned when the Inlet was re-oxygenated in spring. Oxygen levels, therefore, drive the biota in this area, although most crustacea endured stressful levels of oxygen to access the carcasses for much of the time. These data will be valuable in forensic investigations involving submerged bodies, indicating types of water conditions to which the body has been exposed, identifying post-mortem artifacts and providing realistic expectations for recovery divers and families of the deceased.

## Introduction

Terrestrial decomposition and the related taphonomic processes have been, and remain, an area of considerable investigation, see [1]–[3] A solid understanding of decomposition and the biotic and abiotic factors which impact it is valuable not only ecologically, but also in a more pragmatic, medico-legal setting. Much is known in general terms about mammalian decomposition on land, but still there is much to understand since each environment presents a new complexity. However, in general, temporal changes to the body are known and some early temporal statement may be made on elapsed time since death using environmental factors such as insect colonization [2] and plant growth [4]. Marks on the body can be correctly interpreted as post-mortem damage as opposed to mistaken as pre-mortem injury [5], and factors such as whether the remains have decomposed *in situ,* or been moved, or disturbed, can also be determined [6]. However, very little is known about the taphonomy of a body in the marine environment. This study was developed in order to begin an understanding of the decomposition process and the factors that impact it, in a deep coastal marine environment near Vancouver Island, British Columbia.

### Previous Marine Decomposition Studies

The marine taphonomy of extremely large carcasses, such as those of whales, has been studied for over 160 years [7]. Early studies were based on the fortuitous discovery of a whale carcass with no understanding of when the death had taken place [7], but more recently, carcasses of whales and other cetaceans have been deliberately placed in the ocean for study. Most of these studies have been conducted in the deep ocean. Very high species richness has been observed on whale skeletons and these have been compared with assemblages from hot vents [8]. Whale carcasses on the deep sea floor in southern California have been shown to go through three decompositional stages over a long period of time [7]. The first stage has been termed the “mobile-scavenger phase” (p318) where large numbers of vertebrate and invertebrate scavengers remove the majority of the soft tissue within four to eighteen months of death. The second stage is termed the “enrichment-opportunity stage” (p319) which occurs from approximately four months to one and a half years after death in the southern California area. During this stage, very dense assemblages of arthropods, particularly crustaceans, as well as polychaetes colonize the bones and surrounding sediments, which have been enriched by the carcass decomposition [7]. Finally, a sulphur-loving or “sulphophilic stage” (p322) is characterized by a great diversity of anaerobic microbes which feed on the remaining skeleton over decades. In studies in the abyssal region of the north-east Atlantic Ocean, porpoise carcasses were placed at depths of 4000–4800 m at different times and all were completely skeletonized by invertebrates in less than five days [9]. In a later study in the north-east Atlantic Ocean, porpoise carcasses placed at depths of 2555 to 2710 m were observed using a baited camera over a six month period [10]. In the Arabian Sea, two shark carcasses were monitored with a time lapse camera at depths of 1900 and 4040 m [11]. They were only observed for a few days, but in this time, less than a fifth of the tissue was removed by scavengers.

Although such studies of large carcasses are ecologically interesting and do provide some information on the fate of large carcass falls, the size of the animals as well as their very different body type and composition restrict the application of such information for a human death investigation. As well, bodies that are recovered are not usually from such great depths. Therefore, the majority of our knowledge of human marine taphonomy is based on anecdotal reports from individual cases of body recoveries [12]–[14]. These include bodies found floating in the ocean or washed ashore [15]–[17] and bodies recovered from boat or aircraft accidents [18]–[20]. Similarly, microstructural changes to human bones and teeth have been documented in forensic cases from intertidal contexts and from archaeological human remains recovered from shipwrecks [21]–[23]. Although such case histories are extremely valuable, they leave large gaps in our understanding of the parameters which impact marine decomposition. Other studies that may also be usefully referenced are those studies that have examined bone from whale-falls [24], [25] and other fouling of corals and shells created by a range of endoliths [26]. Actualistic and experimental observational studies have also usefully demonstrated the diversity of deep ocean fouling [27].

In an earlier attempt to fill some of these gaps, experiments using pig (*Sus scrofa* L.) carcasses as human proxies were conducted in the shallow coastal marine environment of Howe Sound, near Vancouver, British Columbia [28]–[30]. In those experiments, three freshly killed pig carcasses were deployed in late spring at a depth of 7.6 m and a further three at 15.2 m. Each carcass was separated by at least 150 m and was tethered to a weight by a 2 m rope which allowed it to float or sink, but not drift away. At intervals, the carcasses were examined by divers who observed, photographed and sampled the carcasses until nothing but scattered skeletal remains were present. The experiments were repeated in the fall [28]–[30]. Although valuable data on faunal colonization, decomposition, taphonomic changes and impacts of season and depth, were generated, a limitation of this earlier study, and a continuous problem when conducting research under water, is the lack of carcass accessibility and consequent ability to regularly monitor the carcasses. The research reported here extends and expands the previous pig submersion experiments, and has enabled true real time observational data, collected at per second intervals, to be recovered from a series of three pig carcasses using a range of dynamic sensors and cameras, allowing continuous assessment. The overall objective of this research was to investigate the nature of marine decomposition in pigs (an accepted forensic human proxy) as a continuous, rather than a longitudinal observational study. Continuous studies are by their very nature, data rich, and provide high resolution information, sufficient to capture both sudden and more nuanced environmental effectors.

## Materials and Methods

### Ethics Statement

Simon Fraser University Animal Care Committee permission was obtained to purchase dead pigs. Animal Care Permission #805I-06. The field studies did not involve endangered or protected species. Pigs were euthanized with a humane pin-gun or by electrocution by a licenced butcher and were received after death. No live vertebrates were involved. Carcasses were placed in the ocean. No specific permission required. GPS coordinates Carcass 1–48° 39.0250’N, 123° 29.1423’W, Carcass 2–48° 39.0336’N, 123° 29.1455’W, Carcass 3–48° 39.0650’N, 123° 29.2086’W.

### Research Site

Saanich Inlet is a glacially carved fjord, 24 km long, with a depth of 230 m at its maximum [31]. This inlet is unusual in that it is separated from the more well mixed and oxygenated waters of Georgia Strait by a shallow (70 m), glacial sill which restricts the flow of water into the inlet. The inlet is hypoxic for much of the year, and oxygen is refreshed once a year in the fall [32]. This is a well-studied area and, despite the low oxygen levels, it has high faunal diversity and abundance [33]. It is a popular waterway, close to the metropolitan areas of Greater Victoria and Metro Vancouver, with extensive water use and is the base site for the VENUS (Victoria Underwater Network Under Sea) underwater observatory. The site of the first two carcass placements was at a depth of 95 m and the third was approximately 65 m away at a depth of 99 m. The placement site substrates were fine silt with cobble, over rock (Table 1).

**Table 1:** Carcass deployment.

Parameter	Pig 1	Pig 2	Pig 3
Time of death	1500 h, 5 August 2006	0902 h, 15 Sept. 2007	0800 h, 28 Sept. 2008
Time of submergence	1122 h, 7 August 2006	0800 h, 16 Sept. 2007	0835 h, 29 Sept. 2008
Weight	26 kg	24.7 kg	23 kg
Method of Euthanasia	Electric shock	Pin-gun	Pin-gun
Substrate	Fine silt, 10–20 cm deep, withsome cobble, over rock	Fine silt, 10–20 cm deep,with some cobble, over rock	Fine silt, 10–20 cm deep, with some cobble, over rock, large rocks close by
Location	Longitude(W): 123° 29.1575Latitude(N): 48° 39.0399	Longitude(W): 123° 29.1646Latitude(N): 48° 39.0448	Longitude(W): 123° 29.2069Latitude(N): 48° 39.0829
Depth	95 m	95 m	99 m
Weights	Three weights, linked together	Three independent weights	Three independent weights
Deployment	Dropped over side of boat attachedto an acoustic transponder. Detectedby ROPOS, picked up and placed at site	Deployed by ROPOS	Deployed by ROPOS

### Ocean Network Canada’s VENUS Observatory

The Ocean Network Canada’s VENUS observatory is based out of the University of Victoria, on Vancouver Island. It is a cabled underwater observatory which is designed to deliver high-speed, real-time data to researchers from their experiments on the sea floor [32]. The observatory includes more than 50 oceanographic instruments that gather physical, chemical, acoustic and photographic data continuously. These instruments are connected to the SIIM or Science Instrument Interface Module *via* fiber optic cable and from that to an underwater power and communications hub called the Node. Above the water, the Node connects to one of two shore stations which power the Node and also provides a communication link between the instruments and the University of Victoria. At the university, a Networks Operation Centre (NOC) oversees the functioning of the instruments, and a Data Management and Archive System (DMAS) receives and processes the data. These data can then be accessed worldwide, *via* the internet. Instruments and experiments are deployed using a remotely operated submersible, ROPOS (Remote Operated Platform for Oceanic Science). Instruments are housed on the VENUS Instrument Platform (VIP) and also on the camera tripod. The VIP and camera tripod were linked to the NODE *via* fiber optic cables.

### Ocean Network Canada’s VENUS Observatory Instruments

The instruments are described in Table 2. The camera was used to take both still and video images. The camera (eight mega-pixel Olympus C8080) was housed in copper to prevent fouling and was controlled remotely *via* an internet connection using C-MAP Systems. The camera system included a choice of three 100 W lights with wide, medium and spot reflectors and scaling lasers. The camera could be panned over a 178° arc and tilted over a 90° arc. The camera could be manually or automatically focused and could zoom to take macro images. The camera system was mounted on a tripod approximately 1 m above the carcass, and each carcass was placed between the legs of the tripod.

**Table 2:** Victoria Experimental Network Under Sea (VENUS) instruments used in the study (adapted from www.oceannetworks.ca) (VIP = VENUS Instrument Platform, DCT = Digital Camera Tripod, DCF = Digital Camera Frame).

Carcass	Instrument	Measurements (units)	Frequency	Depth (m)	Location	Distance to carcass (m)
1	Olympus C8080,8 mp camera	Video and still images	Variable	95	48° 39.0250’N, 123° 29.1423’W	1
	Aanderaa Optode4175 S/N18	Dissolved Oxygen (mL/L)Temperature (°C)	60 s	98	48° 39.0719’N, 123° 29.1605’W	89.8
	SeaBird CTD 16plus 4996	Salinity (psu) Density (Kg/m^3^)Conductivity (S/m) Pressure (decibar)	60 s	98	as above	89.8
2	Olympus C8080,8 mp camera	Video and still images	Variable	95	48° 39.0336’N, 123° 29.1455’W	1
	Aanderaa Optode4175 (S/N 579)	Dissolved Oxygen (mL/L)Temperature (°C)	60 s	96	48° 39.0762’N, 123° 29.1690’W	137
	SeaBird CTD 16plus 4997	Salinity (psu) Density (Kg/m^3^)Conductivity (S/m)	60 s	96	as above	137
	Alec ElectronicsCTW 004	Temperature (°C) Conductivity (S/m)	1 s	95	48° 39.0448’N, 123° 29.1646’W	35.3
	AquaDopp CurrentMeter 1176	Pressure (decibar)	60 s	95	as above	35.3
3	Olympus C8080,8 mp camera	Video and still images	Variable	99	48° 39.0650’N, 123° 29.2086’W	1
	Aanderaa Optode4175 (S/N 579)	Dissolved Oxygen (mL/L)	60 s	97.4	48° 39.0707’N, 123° 29.1772’W	39.9
	SeaBird CTD 16plus 4996	Temperature (°C) Salinity (psu)Density (Kg/m^3^) Conductivity (S/m)	60 s	97.4	48° 39.0650’N, 123° 29.2086’W	39.9
	Alec ElectronicsCTW 0003	Temperature (°C)	1 s	99	as above	33.3

Chemical and physical measurements included dissolved oxygen, temperature, salinity, density and pressure. The majority of the instruments were mounted on the VENUS Instrument Platform (VIP) but some were also attached to the camera frame which was linked to the camera tripod. The VIP ranged from 39.9–137 m from the carcasses depending on deployment, and the camera frame ranged from 35.3–39.9 m from the carcass.

### Carcass Deployment

Three pig carcasses were deployed in late summer to early fall over a three year period. Table 1 summarizes the deployments. Pig carcasses were used as human proxies as these have been accepted in forensic entomology research as good models for human decomposition [34]. Also, work in freshwater habitats has shown that submerged pigs decompose similarly to humans in aquatic environments [35]–[37].

Freshly euthanized pigs were transferred to the VENUS research vessel, and then to the marine deposition site. The carcasses were normally refrigerated overnight and deployed the next day, although a camera problem with Carcass 1 resulted in a delay of 20 h. All carcasses were in rigor, with lividity when they were deployed. The carcasses were weighted to keep them in view of the camera, and were deployed using a remotely operated submersible: Remote Operated Platform for Oceanic Science (ROPOS).

Carcasses were weighted before deployment to keep them within camera range. Although bloat does not occur at this depth [13], animal activity could easily move the carcasses out of range. Weights on Carcass 1 were linked by rope to form a “handle” over the torso for ROPOS to move the carcass to the site. Carcass 1 was weighted then attached to a transponder and dropped over the side of the research vessel, close to the camera tripod. ROPOS then located the transponder and carried the carcass to the pre-established camera tripod and then, guided by the first author, placed the carcass directly under the tripod, approximately 1 meter under the camera itself. This weighting pattern was not optimal, and Carcass 2 and 3 were weighted with separate weights at three areas of the body, neck, shoulder and groin, and ROPOS carried the carcasses from the ship’s deck to the sea floor exposure site.

Once the carcasses were in position, they were not disturbed or physically accessed by humans until the experiment was terminated. The day of submergence was listed as Day 0.

### Observations

Each carcass was observed and photographed during and immediately subsequent to placement and, from then on, several times a day for a period of approximately 30 minutes at a time. Each session was recorded by video in its entirety and still images were taken at will. Observation periods were kept to a minimum to avoid unnecessary light pollution. Observations were usually made at 0800 h and 1900 h Pacific local time, although observations were also made at 2200 h and 0400 h on occasions.

## Results

In all three cases, it was anticipated that the carcasses would be observed until no soft tissue remained. However, Carcass 1 was dragged from camera range after Day 23, so could not be observed after this time. Carcass 2 was observed until all soft tissue and cartilage had been removed. Carcass 3 was observed for 135 days post submergence, when the experiment was terminated.

At these depths, there is no visible light and any observations required the camera lights to be turned on briefly. It was anticipated that the lights would impact the fauna and either attract or repel different species as earlier experiments using divers with lights showed that many animals were attracted to the lights [30]. Some animals would suddenly disperse when the lights were first turned on, but ventured back very rapidly and zooplankton were attracted in large numbers although most fauna did not appear unduly affected by the light. Nevertheless, lighting was kept to a minimum.

In all cases, the carcass biomass was removed due to arthropod scavenging activity, with no classic signs of decomposition visible (such as bloat, putrefaction, skin slippage, active and advanced decay).

### Faunal Colonization and Scavenging

**Figure 1:**
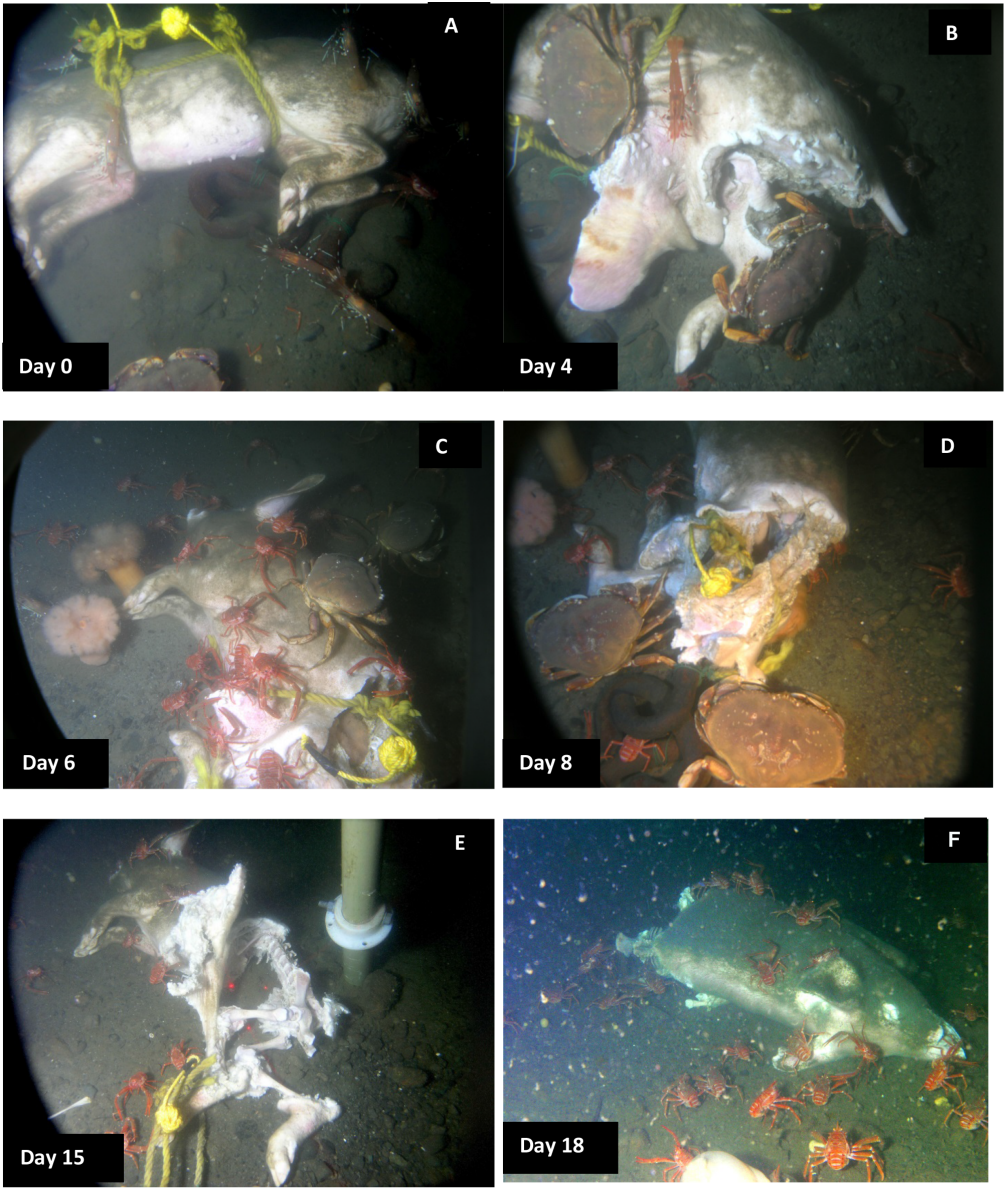
Progression of carcass scavenging and degradation for Carcass 1, 2006. A. Carcass first placed, *Pandalus platyceros* Brandt (three spot shrimp) (*P.p.*) and *Metacarcinus magister* Dana (Dungeness crab) *(M.m.)* immediately attracted; B. Shark wound extremely attractive to all fauna; C. Intestines exposed, many *M.m.* and *Munida quadrispina* Benedict (squat lobster) (*M.q.*) feeding; D. Spinal column exposed, organs removed; E. Carcass dragged from weights and away from camera, much of carcass skeletonized, lasers indicate 10 cm; F. Carcass turned 180° by fauna, head area mostly intact with some grazing marks from *M.q.* (Ocean Network Canada’s VENUS observatory).

**Figure 2:**
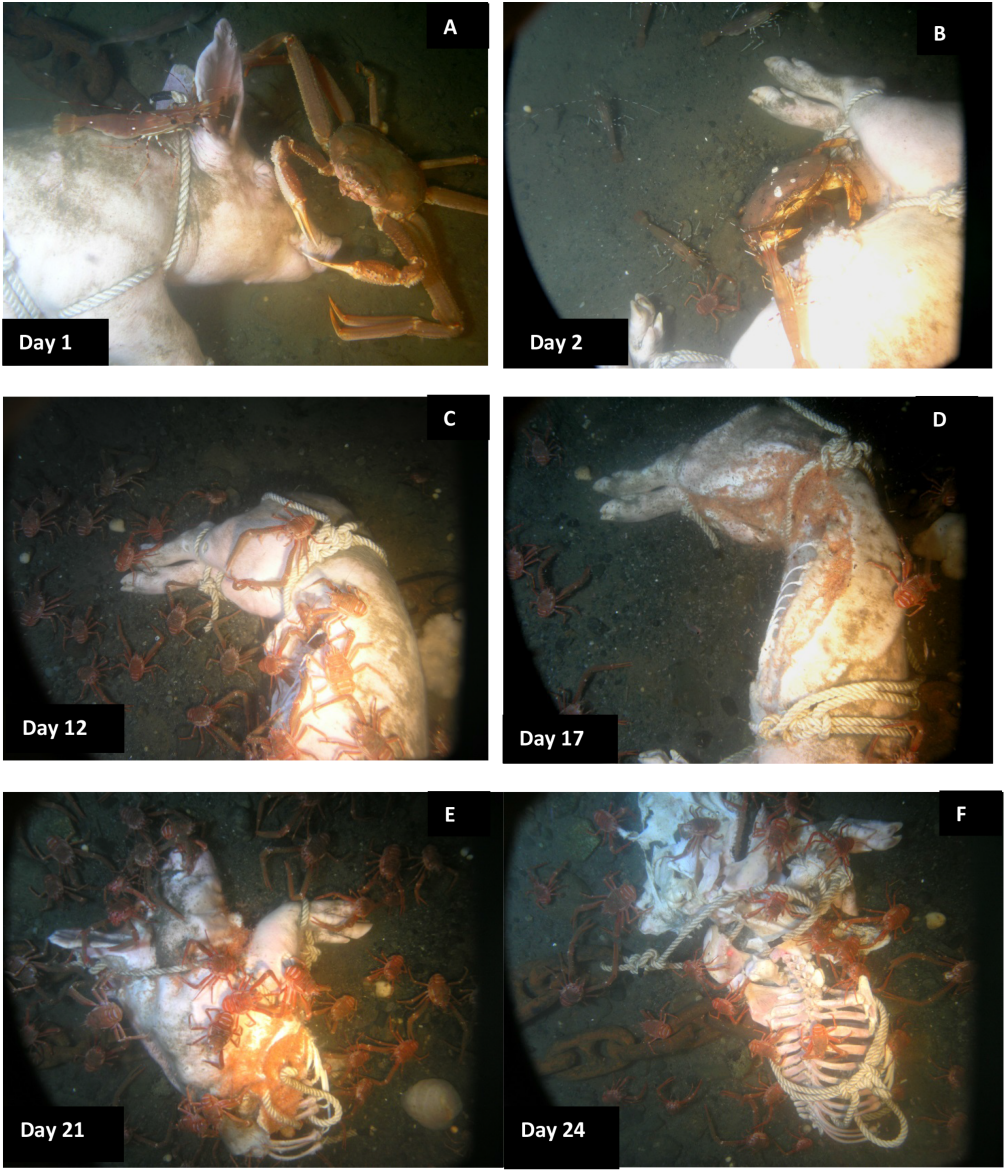
Progression of carcass scavenging and degradation for Carcass 2, 2007. A. *Chionectes tanneri* Rathbun (tanner crab) attracted to the face; B. *Metacarcinus magister* Dana (Dungeness crab) *(M.m.)* reaching into abdominal area and consuming internal tissues with *Munida quadrispina* Benedict (squat lobster) (*M.q.)* and *Pandalus platyceros* Brandt (three spot shrimp) *(P.p.)* waiting nearby; C. Rib ends exposed and large numbers of *M.q.* dominate the carcass; D. *Orchomenella obtusa* Sars (*O.o.*) cover the exposed tissue; E. Half of carcass removed by shark, carcass being skeletonised from inside out by *O.o.* with *M.q.* feeding on skin; F. Skin pulled over torso and cranium by *M.q.* exposing skeleton (Ocean Network Canada’s VENUS observatory).

**Figure 3:**
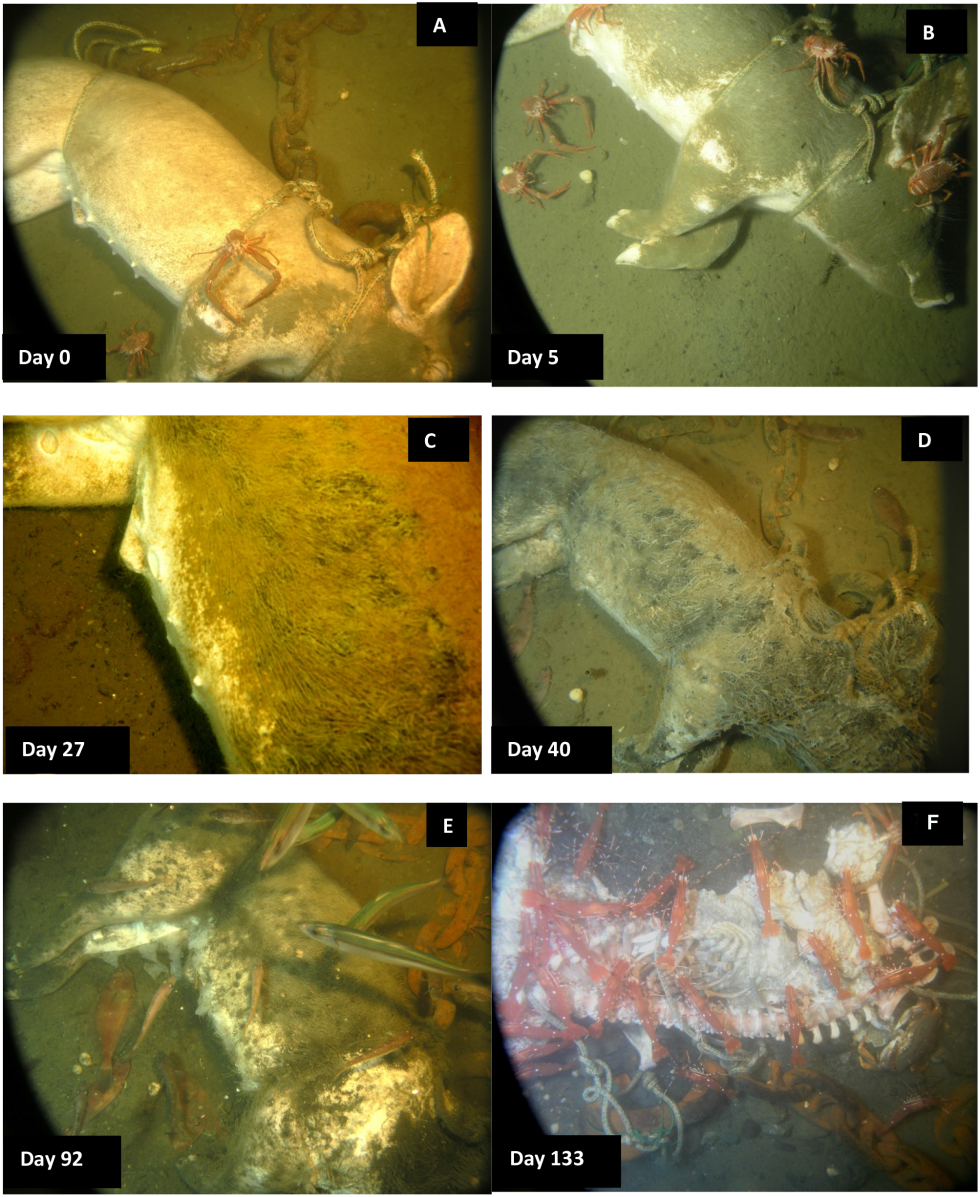
Progression of carcass scavenging and degradation for Carcass 3 2008/2009. A. A few *Munida quadrispina* Benedict (squat lobster) *(M.q.)* attracted, but very few fauna present; B. Silt covering carcass and only a few *M.q.* present, but no damage visible. C. Some grazing marks in groin area from *M.q.* but skin not broken through; D. Bacterial mat forming over entire carcass. Note numerous *Lyopsetta exilis* (Jordan & Gilbert) (slender sole) on substrate; E. Sudden influx of large number of fish; F. Large numbers of *Pandalus platyceros* Brandt (three spot shrimp) as well as *Metacarcinus magister* Dana (Dungeness crab) with very few *M.q.* (Ocean Network Canada’s VENUS observatory).

Tables 3 and 4 tabulate the decomposition and fauna of all three carcasses as well as dissolved oxygen levels. Figures 1A–F document the progression of decomposition and scavenging of Carcass 1 which was observed from 7–30 August 2006; Figures 2A–F document the progression of Carcass 2, which was observed from 16 September −27 November 2007, and Figures 3A–F document the progression of Carcass 3, which was observed from 29 September 2008–11 February 2009.

**Table 3:** Comparison of decomposition of all three carcasses over time.

*Day*	*Carcass 1*	*Carcass 2*	*Carcass 3*
0	Carcass fresh, in rigor, lividity fixed.Sank immediately.	Carcass fresh, in rigor, lividity set, somegreenish discoloration in abdominal area.Sank immediately.	Carcass fresh, in rigor, lividity set. Sank immediately.
1	Silt on carcass, Carcass being rocked byanimals.	Greenish discoloration of abdomen morepronounced. Rip in skin approximately7 cm long, in abdominal/groin area.	No change
2	Carcass moved 180° and 1.5 m fromoriginal site. Large piece of flesh tornfrom hind quarters by large animal andlarge flap of tissue area pulled awayfrom abdominal region.	Adipose tissue bulging from abdominalopening. Opening now about 10 cm long.Signs of grazing in face and ears. No sign ofbloat. Small circular marks in skin aboveabdominal rip, caused by crab claws. Smallgrazed area on shoulder. Gut coil pulled outby *M.m.*	No change, silt depositing on carcass
5	Large amount of tissue removed fromhind quarters. Abdominal cavity openedand intestines and internal organs visible.Lower spinal column exposed. Very littlefeeding damage to head. No outward signsof decomposition, just tissue removal. Leftrear leg partially de-fleshed	Grazing marks all around snout into lip.Eyelid intact. Edge of ear grazed. Abdomencaved in slightly. Small circular artifacts onside of carcass left by *M.m. M.m.* seenremoving tongue day before.	No change, more silt on carcass.
6	Large flap of tissue still present. Carcasshas been moved further 15 cm.	No tissue protruding from abdomen. Feedingmarks on edges of ears. *M.m.* reaching deepinto abdominal area.	No sign of any damage to tissue.
8	Carcass moved three times over the day,60–80 cm from previous day.	Large amount of tissue pulled out ofabdominal area. Opening much enlarged.Grazing marks on rear hocks and face. *M.m.*artifacts picked at by *P.p.*, so less distinct.	Carcass still completely intact, no damage visible.
10	Back legs partially eaten, hind quartersalmost gone. Most of lower internal organsgone. Rear part of carcass skeletonized.Some tissue adhering to bones. Weightsholding carcass under camera interlinked soslipped off once rear part of carcass gone.Carcass moved another 30–40 cm. Lowerribs exposed. Upper body seems intact.	Abdominal opening now from groin tosternum, and some skin and tissue removedto leave large opening. Skin removed andfeeding occurring between back legs. Skinbeing removed to open abdomen up further.Eye is gone. Part of ear gone.	Carcass intact, no damage.
12	Further movement of carcass. Upper bodystill intact, rear area mostly skeletonized,but cartilage still present.	Six ribs visible and skeletonized. Abdomenis now large open hole. Opened area nowextends down back legs and almost up tofront legs. Thoracic cavity open, organsvisible. Round grazing areas in severalareas of the skin caused by *M.q.*	Carcass intact, no damage. Some silt displaced on front leg.
13	Extensive damage to abdominal area, upperbody still intact, but seems that organs havebeen removed. Back legs still articulated.Carcass has been pulled free of all weightsand moved further from camera range.	More ribs exposed and fatty tissue underskin grazed back further around abdominalarea. Abdomen completely open.	Carcass intact, no damage.
14	Carcass pulled further out of range. Piece oftissue has been torn from stomach area andis being eaten.	Seven ribs visible. Skin further removedalong abdomen walls, exposing muscletissue. Grazing damage around face.	Possibly, very slight feeding damage to one nipple.
15	Carcass pulled further away from camera,outside tripod area. Grazing damage to snoutarea. Flap of skin and muscle pulled backfrom torso. One bone separated from bodyin morning observation, but entire hind legdisarticulated by evening observation, andcarcass dragged another ∼30 cm. Twolowest ribs visible and skeletonized.	Skin removed from between back legs tofront legs and up to mid line of carcass.More ribs exposed but muscle tissue stillremaining on side. Bulk of carcass stillintact. Only slight grazing at head area.Cartilage still present on ribs.	No change.
16	Carcass has been completely turned aroundso that head now faces camera. Snout grazedand some nasal bones visible. Eye socket empty,grazed area behind ears. Lower part of carcass,including pelvis, missing.	Entire abdominal area open, organs appeargone. Can still see hairs on pig skin in headand body area. Tissue still present in eyesocket. Grazed tissue around abdominalopening has now been pierced.	Not observed.
17	Carcass has been moved again. Much ofsnout skeletonized. Disarticulated foot.	All exposed muscle tissue covered in *O.s.*Muscle tissue still visible from lower part ofback legs to front legs. Ribs exposed. Mostof tissue gone in eye socket.	Silt on face blackening
18	Disarticulated foot just bones now. Most of front half of body still intact.	Rear half of carcass has been completelyremoved. Skin is ‘rucked’ up, exposing ribsand sternum. Different weighting allowsfront portion of remains to stay in cameraview. Part of upper lip gone. Rest of pigcompletely gone from camera range. Hairstill present on skin of upper part of pig.	Dark film, bacterial mat, on hair on head area and front legs.
20	Carcass moved further from camera. Hasbeen turned around again. No organs visiblein body cavity. Upper body still mostly intact,but hollowed out. Much of spinal columncompletely skeletonized. Muscle tissue andskin still present on front portion of body.	Skin of upper body pulled up partially as ifit were a shirt. Skin seems a bit loose overupper body, neck and head as if less tissueunderneath. Head area still intact, althoughskin slightly loose around jowls. Circulargrazed area on front leg from *M.q.*	Bacterial mat thicker on head, and now on back.
22	Carcass being moved constantly. Majority ofcarcass is almost out of sight of camera. Hindleg in two pieces	Skin is loose and wrinkled on bones of frontof carcass and all head area as if all softtissue beneath it has been removed. Lips andeyes gone. Round grazed areas in skin onfront leg. By end of day, skin being pulledup and exposing ribs. Cartilage still present.	A few small grazing marks at nipples and inside of left rear leg.
23	Carcass dragged from camera range.No further observations	Skin being dragged up over head by animals,exposing clean bones. Skin like a loose bagover head. Rib cage completely exposed andarticulated, cartilage present. Large holes inskin. Skin is being pulled in all directions by*M.q.* Mandible disarticulated but in position.Scapula has been pulled free.	Another small grazing mark between back legs noted.
24	-	Skin has been pulled over top of head. Verylittle skin remaining.	Not observed
26	-	Last piece of skin remaining is ears. Allbones remain *in situ* and are clean of allvisible soft tissue. Cartilage present. Ribcage intact	Not observed
27		Rib cage still articulated, Only soft tissueleft is the ears.	Two grazed areas apparent in skin in groin region but damage is very shallow and does not break into abdomen
30	-	Small pieces of ears still remaining,cartilage still present, almost all ribscollapsed. Sternum still intact.	Grazed areas in groin area do not penetrate abdominal wall. Dark grey/black bacterial mat on silt all over carcass except between back legs and along abdomen where *M.q.* have been grazing
33	-	All ribs separated from spinal column.	No change
38	-	All cartilage seems to have been removed	Bacterial mat getting thicker
46	-	(Day 47) Bones looking black/dark greyin places. Spinal column still intact	Bacterial mat thickening and parts sloughed off by *L.e.*
67	-	(Day 71) Bones all covered in black/greyfilm	Film on carcass thicker, no further grazing occurring, carcass being obscured by bacterial mat, reddish area at centre of chest
92	-		Areas of bacterial mat removed from carcass
98	-		Large white areas of skin exposed, evidence of feeding at abdomen and tissue looks grey in places
106	-		Entire exposed shoulder area and areas of abdomen, legs and rump, has been opened up and muscle tissue exposed, putty colored
125	-		Partial skeletonization
135	-		Some disarticulation, and skeletonization. Study terminated.

*M.m. = Metacarcinus ( = Cancer) magister* Dana, *M.q. = Munida quadrispina* Benedict, *O.s. = Orchomenella obtusa* Sars, *L.e. = Lyopsetta exilis* (Jordan & Gilbert).

**Table 4:** Comparison of faunal scavenging of all three carcasses over time, together with dissolved oxygen levels.

*Day*	*Carcass 1*	*Carcass 2*	*Carcass 3*
0	Large numbers of *M.q*. attracted immediately.Also some *M.m.* and *P.p.* attracted. All pickingall over carcass, particularly at face.**Oxygen = 1.4 mL/L**	Many *M.q.* immediately attracted, within hours,many *M.m.* and *P.p.* also present. All appear tobe picking all over carcass.**Oxygen = 0.9 mL/L**	A few *M.q.* present. Lots of herring present. *M.q.* picking at nostrils. *L.e.* on substrate.**Oxygen = 0.5 mL/L**
1	Many herring present, also *M.q.*, *P.p.* also*M.m.* **Oxygen = 0.7 mL/L**	*P.p.* picking at eye. Some *M.m.* present, one seenreaching into abdominal cavity through rip. Single*C.t.* present. All animals dispersed at first whenlights turned on, then immediately returned.**Oxygen = 0.9 mL/L**	Very little activity. A few *M.q.* present. Little interest in carcass. No *M.m.* or *P.p.* **Oxygen = 0.5 mL/L**
2	Large scavenger not observed but believed tobe *H.g.* All arthropod scavenger activity focusedon bite site from this time onwards. Mainscavengers are large numbers of *M.q.* Severalfish species swimming around but not attractedby carcass. **Oxygen = 1.0 mL/L**	Several *M.m.* present, many *P.p. M.m.* feeding onskin and underlying tissue, *P.p.* feeding on extrudedtissue. *M.m.* ate all tissue bulging out in a fewminutes. Several *M.m.* reaching into abdomen andpulling large chunks of tissue out to eat. A few *M.q.*present. Many fish swimming through area. Manyzoo-plankton, particularly *S.e.* often obscuring view.Not interested in carcass. Probably attracted by lights.**Oxygen = 0.9 mL/L**	A few *M.q.* on and around carcass. *L.e.* on substrate. Lots of small euphausids.**Oxygen = 0.5 mL/L**
5	Two *M.m.* feeding at new wound area. Several*M.q.* and *P.p.* on substrate around carcass andon carcass, feeding. **Oxygen = 1.0 mL/L**	Fauna acclimatized to lights, Many *P.p.*, manyzoo-plankton, Several *M.m.*, same as before, can beidentified by barnacle pattern on carapace. Some*M.q. M.m.* feeding at abdomen. *M.m.* and *P.p.*feeding at anus. *P.p.* also feeding at eye and mouth.**Oxygen = 0.8 mL/L**	Only a few *M.q.* on carcass and substrate and a few *L.e.* **Oxygen = 0.4 mL/L**
6	Large numbers of *M.q.*, *P.p.* and *M.m. M.m.*capable of rocking the entire carcass. Camerascans of area show large numbers of thesespecies actively moving towards carcass.Most activity at bite site. Also some *M.q.*picking at face. Other species such as *P.h.*and *O.r.* briefly visited carcass.**Oxygen = 1.0 mL/L**	Many *M.q.*, *M.m.* and *P.p.* present. *M.m.* ripping atstomach area and reaching into abdominal cavity.*M.m.* also attempting to catch *M.q.* and a fish. Many*S.e.* suddenly appeared, attracted to lights, obscuringcarcass at times. Many herring present. Not interestedin carcass. **Oxygen = 0.8 mL/L**	A few *M.q.* around. Many euphausids or *S.e.* suddenly appeared in a cloud **Oxygen = 0.4 mL/L**
8	Many *M.q.* all around carcass and feeding. Several *M.m.* feeding on carcass, rockingit and almost rolling it over. *P.p.* feedingand on substrate surrounding carcass.**Oxygen = 1.0 mL/L**	Many *M.q.*, *M.m. P.p* feeding and *S.e.* present. *M.m.*and *P.p.* pulling adipose tissue and possibly lung outof abdominal area and feeding. Most *M.q.* stayingaway from *M.m.* One *M.m.* feeding on *M.q.* **Oxygen = 0.7 mL/L**	Several *M.q.* on and around carcass **Oxygen = 0.3 mL/L**
10	*M.m.* seen moving carcass, almost rollingit over at times. Lots of *M.q.* and *P.p.* feeding,picking at skin and intestines. *M.m.* also feedingon *M.q.* as well as feeding on and mostly insidecarcass, reaching inside carcass to pull out organsand tissue. Some feeding also at head end by *P.p.*and *M.q.* All three species present day or night. **Oxygen = 0.9 mL/L**	*M.q.* pulling pieces of skin and tissue fromedge of abdominal area. Many *M.q.* all overcarcass, no *M.m.* or *P.p.* on carcass or in vicinity.**Oxygen = 0.6 mL/L**	A few *M.q.* on carcass, picking, but not causing damage to skin.**Oxygen = 0.3 mL/L**
12	Many *M.q.* feeding all over carcass. No *M.m.*or *P.p.* **Oxygen = 0.9 mL/L**	Many *M.q.* present, and feeding over body, mostlyat abdomen and head area. *M.q.* pulling strands oftissue or organs from abdomen. A few *M.m.* and*P.p.* seen in area. **Oxygen = 0.6 mL/L**	A few *M.q.* on and around carcass**Oxygen = 0.2 mL/L**
13	Small red amphipods, *O.s.* feeding at exposedtissue at the edge of the opening. *M.m.* feedingin abdominal area and hind area. Many *M.q.*feeding but no *P.p.* present. **Oxygen = 0.8 mL/L**	Many *M.q.* and several *M.m.* feeding on carcass.Some *M.m.* are recurring specimens. *M.m.* reachinginto abdominal opening. **Oxygen = 0.7 mL/L**	Single *M.q.* on carcass, a couple more on substrate. **Oxygen = 0.2 mL/L**
14	*M.m.* and *M.q.* feeding on carcass and resting onsubstrate. *L.e.* on substrate nearby. Dogfish andmany smaller fish swimming over. No *P.p.* Just afew *O.s.* on exposed skin. **Oxygen = 0.7 mL/L**	Many *M.q.* present and some *P.p.* First appearanceof a few *O.s.* on exposed flesh where skin removed.Several *M.m.* on and around carcass.**Oxygen = 0.6 mL/L**	Single *M.q.* on carcass, a couple more on substrate. **Oxygen = 0.2 mL/L**
15	Fewer animals present. Almost entirely *M.q.* witha few *M.m.* nearby. No *P.p.* **Oxygen = 0.5 mL/L**	Many *P.p.* and *M.q.* feeding all over abdomen andhead. *M.q.* at abdomen as no *M.m.* present. Pullingpieces of tissue off to feed. **Oxygen = 0.6 mL/L**	Several *M.q.* on carcass **Oxygen = 0.2 mL/L**
16	Many *M.q.* present and feeding. Also, many *M.q.*in vicinity of carcass. No *M.m.* or *P.p.* **Oxygen = 0.5 mL/L**	Many *M.q.* feeding all over body and some resting onbody. Some *P.p.* but no *M.m.* **Oxygen = 0.6 mL/L**	Not observed **Oxygen = 0.2 mL/L**
17	Many *M.q.* on substrate and around disarticulatedfoot. A *M.m.* at foot but no *P.p.* **Oxygen = 0.5 mL/L**	Sudden presence of very large numbers of *O.s.*covering all tissue where skin has been removed.*M.q.* inside gut area. perforations being created inmuscle tissue from inside. Large numbers of *M.q*,*P.p.* and *O.s.* present. **Oxygen = 0.7 mL/L**	No visible fauna. **Oxygen = 0.2 mL/L**
18	Many M.q. on carcass and substrate but no M.m.or P.p. **Oxygen = 0.7 mL/L**	Many M.q. present all over upper part of body.A few O.s. present. Animal that removed lowerpart of carcass unknown, but probably H.g.**Oxygen = 0.6 mL/L**	A few M.q. picking at groin area **Oxygen = 0.3 mL/L**
20	Many *M.q.* feeding on body. Single *M.m.* close bybut not feeding. No *P.p.* or *O.s.* observed. Manysmall fish present but no interest in carcass.**Oxygen = 0.8 mL/L**	Many *O.s.* covering exposed tissue and also inneck region. Many *M.q.* and some *M.m.* present.**Oxygen = 0.5 mL/L**	No fauna on carcass **Oxygen = 0.2 mL/L**
22	Several *M.m.* in area, and feeding as well as *M.q*. feeding on carcass and parts, as well as restingon substrate. **Oxygen = 0.7 mL/L**	Many *O.s.* at throat area and on ropes. Very largenumbers of *M.q.* on and around carcass and only asingle *M.m.* **Oxygen = 0.4 mL/L**	No fauna on carcass, a *M.q.* nearby on substrate. **Oxygen = 0.3 mL/L**
23	A few *M.q.* on silt. Carcass no longer in sight.No further observations.	Many *M.q.* all over remains of carcass and onsubstrate, a few *O.s.* **Oxygen = 0.4 mL/L**	No fauna carcass, but a *M.q.* on substrate, and an *L.e.* **Oxygen = 0.3 mL/L**
24	-	*M.q.* and *P.p. M.q.* pulling at skin and eating it.**Oxygen = 0.5 mL/L**	Not observed **Oxygen = 0.3 mL/L**
26	-	*M.q.* feeding and pulling at remains of skin.**Oxygen = 0.5 mL/L**	Not observed **Oxygen = 0.3 mL/L**
27	-	Many *M.q.* feeding on remaining bits of skin.No other arthropods present. **Oxygen = 0.5 mL/L**	A few *M.q.* and *L.e.* in area but not on carcass. **Oxygen = 0.3 mL/L**
30	-	Only *M.q.* picking at bones. Bones beingconstantly moved by M.q. **Oxygen = 0.4 mL/L**	A few *M.q.* on substrate. **Oxygen = 0.3 mL/L**
33	-	*M.q.* moving bones, picking at cartilage.**Oxygen = 0.4 mL/L**	Many *L.e.* on substrate, nothing on carcass. No *M.q.* around. **Oxygen = 0.2 mL/L**
38	-	Some *M.q.* present on remaining bones.**Oxygen = 0.4 mL/L**	A single *L.e.* on substrate, nothing on carcass. No *M.q.* around. **Oxygen = 0.2 mL/L**
46	-	(Day 47) Some *M.q.* on bones and substrate**Oxygen = 0.3 mL/L.**	A few *M.q.* and *L.e.* in area. A few immature *M.q.* observed in sand coming out of a hole **Oxygen = 0.4 mL/L**
67	-	(Day 71) A few *M.q.* near bones.	One or two *M.q.* on substrate. **Oxygen = 0.4 mL/L**
92	-	-	Large numbers of fish present, swimming over the carcass but no arthropods. **Oxygen = 0.8 mL/L**
98	-	-	Several *M.q.* on body as well as a few *P.p.* A few fish present. **Oxygen = 1.1 mL/L**
106	-	-	Large numbers of *P.p.* and some *M.m.* and *M.q.* feeding on carcass and removing tissue. **Oxygen = 1.9 mL/L**
125	-	-	Many *P.p.* and some *M.m.* **Oxygen = 1.6 mL/L**
135	-	-	Many *P.p.* and some *M.m.* **Oxygen = 1.8 mL/L**

*M.m. = Metacarcinus* ( = Cancer) magister Dana, *M.q.* = *Munida quadrispina* (Brandt), *P.p.* = *Pandalus platyceros, H, g.* = *Hexanchus griseus Bonneterre, P.h.* = *Pycnopodia helianthoides* (Brandt), *O.r.* = *Octopus rubescens* Berry, *O.s.* = *Orchomenella obtusa* Sars, *L.e.* = *Lyopsetta exilis* (Jordan & Gilbert), S.e. = *Sagitta elegans* Verrill.

**Figure 4:**
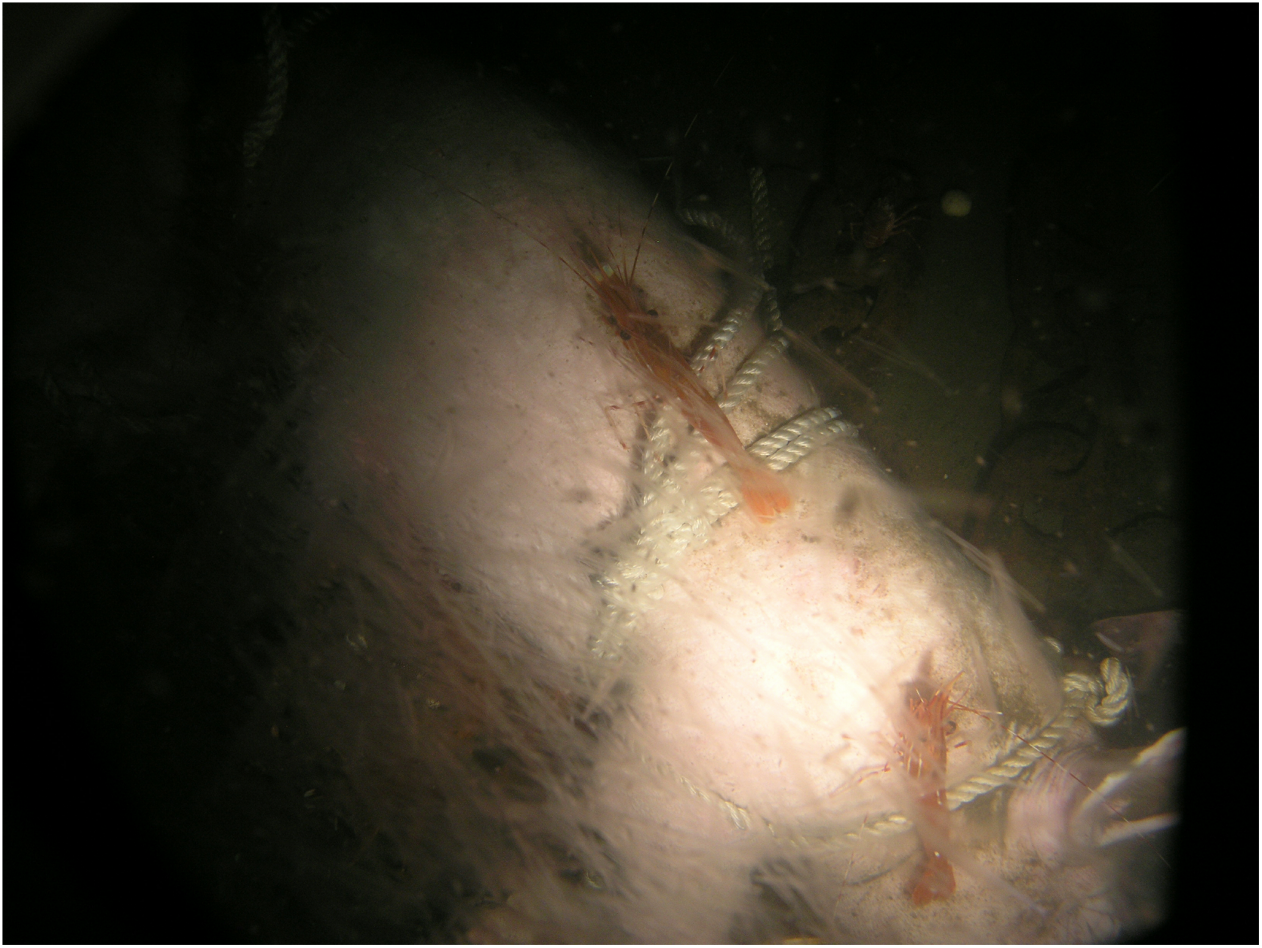
*Sagitta elegans* Verrill (arrow worms) and other plankton attracted by the lights on Carcass 2, on Day 2. *Pandalus platyceros* Brandt (three spot shrimp) on carcass (Ocean Network Canada’s VENUS observatory).

**Figure 5:**
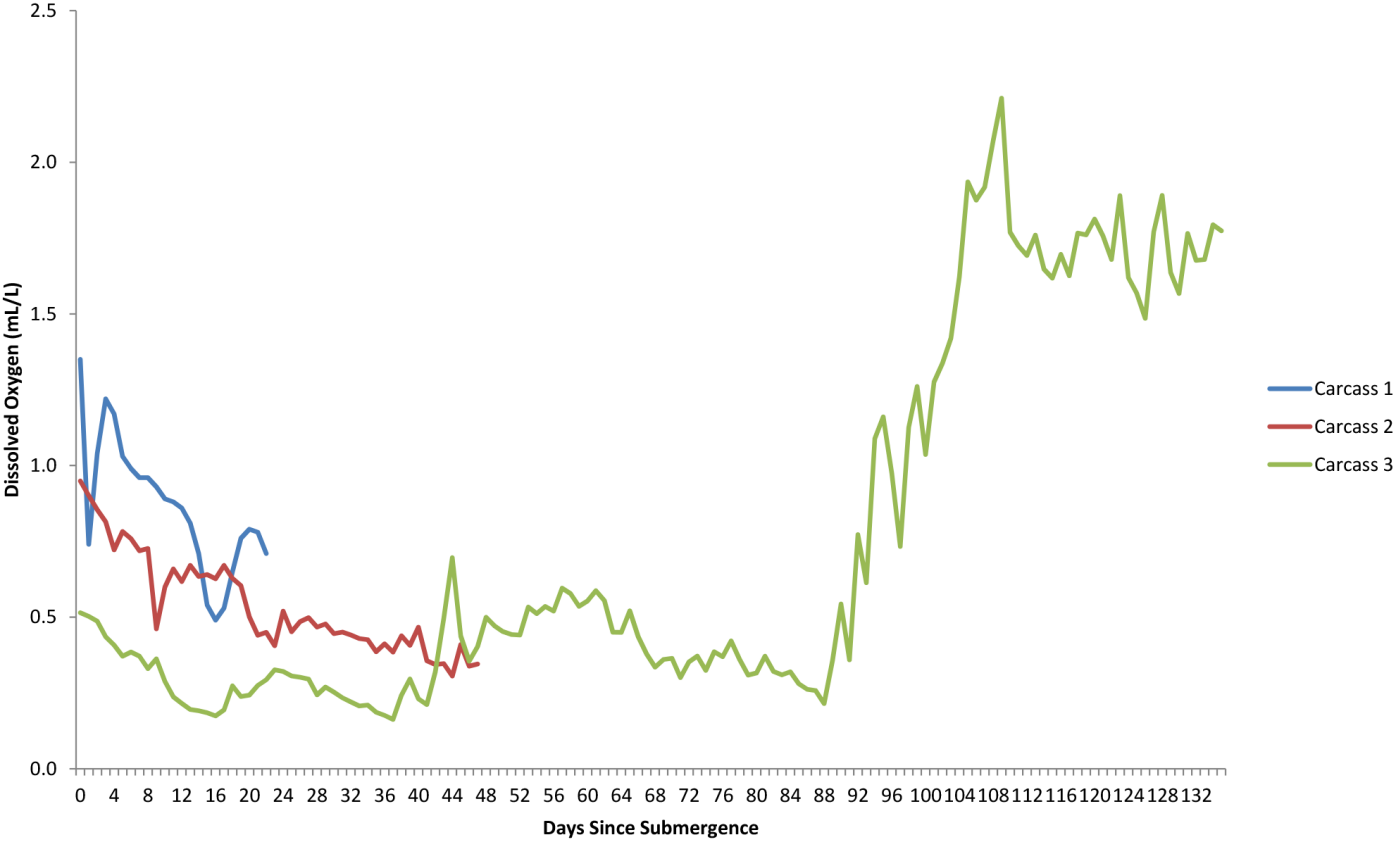
Dissolved oxygen (mL/L) for the duration of study for each carcass. Oxygen measured using Aanderaa Optode 4175 every 60 s. (Ocean Network Canada’s VENUS observatory).

Within minutes of placement, large numbers of *Munida quadrispina* Benedict (squat lobsters, Family Galatheidae) arrived at Carcass 1 and 2 and began to pick at the skin, attracted to the entire carcass, with some preference for the orifices (Video S1). Scanning the camera around the area showed that very large numbers of *M. quadrispina* were actively moving towards the carcasses from all areas. A large herring ball (*Clupea* sp. Family Clupeidae) was present when Carcass 1 was deployed, but although the fish swam over the carcass repeatedly, they showed no direct interest. *Pandalus platyceros* Brandt, (Three Spot Shrimp, Family Pandalidae), the largest of the local shrimp, and *Metacarcinus* ( = *Cancer) magister* Dana (Dungeness crabs, Family Cancridae) were also attracted immediately and picked at the carcasses (Figure 1A). Carcass 2 also attracted a tanner crab (*Chionectes tanneri* Rathbun) which was observed picking at the facial area (Figure 2A). When the lights were first turned on at the start of the studies, some of the larger crustaceans were repelled by the lights but returned in seconds, and after two days, they were no longer affected. Many zooplankton were present, probably attracted to the lights. These were particularly noticeable above Carcass 2, with large numbers of smaller zooplankton, including arrow worms ((*Sagitta elegans* Verrill, Phylum Chaetognatha, Order Aphragmophora, Family Sagittidae) which were sometimes so numerous that they obscured the carcass from view (Figure 4). Carcass 3 was also immediately attractive to *M. quadrispina* but dramatically fewer specimens arrived, picking at the nasal orifices and overall carcass. No *M. magister* or *P. platyceros* were attracted (Figure 3A). Dissolved oxygen levels were 1.4 mL/L when Carcass 1 was deployed, 0.9 mL/L when Carcass 2 was deployed and 0.5 mL/L when Carcass 3 was deployed (Figure 5).

**Figure 6:**
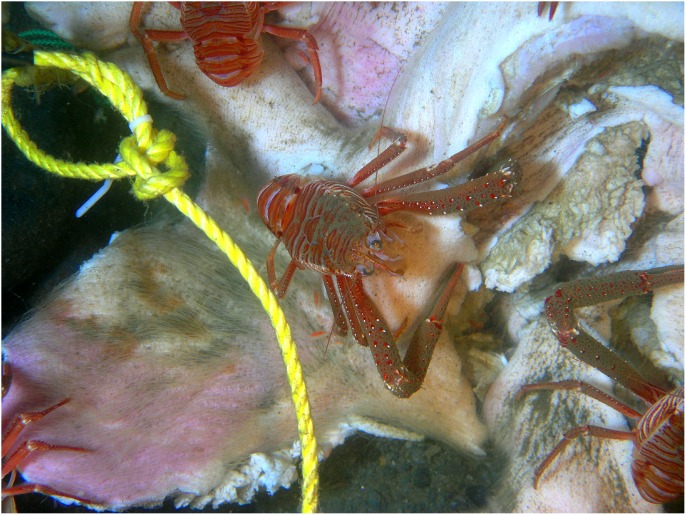
*Munida quadrispina* Benedict (squat lobster) picking at the damaged area of the abdomen of Carcass 1 on Day 2 (Ocean Network Canada’s VENUS observatory).

On Day 2, a substantial portion of the rump area of Carcass 1 was removed, and a large flap of skin and flesh from the abdominal area was opened. The carcass had been moved approximately 1.5 meters to a location 180° from its original site, to the other side of the tripod area. The tissue appeared to have been avulsed due to a large bite and the pattern, shape and size of the bite suggested that it had been caused by a blunt-nose sixgill shark (*Hexanchus griseus* Bonneterre) [38]. The pattern of the bite mark suggested a single bite. No shark activity was observed and no further damage occurred to Carcass 1, but the damage had a major impact on the future faunal scavenging of Carcass 1 as all crustacean activity became focused on this site, with very little activity seen at the orifices. *Metacarcinus magister* and *P. platyceros* fed at the wound site (Figure 1B), as well as large numbers of *M. quadrispina* (Figure 6). On Day 2, Carcass 2 was still intact but a 7 cm rip was seen in the lower abdominal area, with small marks above the rip, probably caused by the larger crabs anchoring themselves when feeding at the abdominal rip or from the picking action of the chelicerae (Figure 2B). *Metacarcinus magister* were seen reaching deeply into the abdominal rip and pulling out tissue. Feeding activity occurred all over Carcass 2, with *M. magister* and *P. platyceros* feeding at the abdominal area as well as the head. *Pandalus platyceros and M. quadrispina* fed all over the body, but did not appear able to break into the carcass without the large crab activity. Adipose tissue that bulged out of the abdominal opening was very rapidly consumed. The main fauna directly feeding on the carcass were *M. magister*, *P. platyceros* and *M. quadrispina* with a variety of fish and large numbers of plankton swimming over. Several large *M. magister* fed constantly on the carcass, regardless of time of day. The large crabs were distinctive due to the patterns of barnacles on their carapaces so individuals could be identified. The same crabs stayed at the carcass to feed and were seen actively ripping large quantities of tissue from inside the abdomen. New crabs continued to arrive. When the larger crabs moved away from the abdomen or to a different region of the body, *M. quadrispina* and *P. platyceros* would immediately move in to feed, with *M. quadrispina* sometimes entering the body cavity but, when *M. magister* was feeding at the abdominal area, the smaller crustaceans would move to feed at the head or rump area in active avoidance of the larger crabs, which would sometimes grab at them and were seen to feed on them. On occasions, *M. magister* would fight amongst themselves over tissue or a *M. quadrispina*. In contrast, at this time, very little activity was observed at Carcass 3, with only one or two *M. quadrispina* on and around the carcass and fish such as *Lyopsetta exilis* (Jordan & Gilbert) (slender sole) swimming over, and sometimes resting on the carcass but otherwise showing little direct interest. The carcass began to be covered with fine silt (Figure 3B). Dissolved oxygen levels over Day 1 and 2 were 0.7–1 mL/L for Carcasses 1 and 2 but remained at 0.5 mL/L for Carcass 3.

Over the subsequent days, both Carcass 1 and Carcass 2 were scavenged by *M. quadrispina, M. magister* and *P. platyceros* (Video S2 and S3). *Munida quadrispina* fed at the wound site, the flap of skin and somewhat at the facial orifices of Carcass 1 and at the face and anus of Carcass 2, as well as at the abdominal area when *M. magister* were not present. Several large *M. magister* fed at the wound site of Carcass 1 and the abdominal area of Carcass 2 (Video S4) and entered the abdominal area once enough tissue was removed. They then proceeded to eat the internal organs and tissues. Their activities alone were enough to lift and move the entire carcass (Video S5). Pieces of tissue dropped by *M. magister* would be rapidly picked up by *M. quadrispina*, although *M. quadrispina* also fed constantly on the carcasses directly. However, at sites where *M. quadrispina* alone were feeding, the skin was only grazed, not broken as they appeared to require the larger crabs to break through before they could feed on the internal tissue. Fish, such as herring, dogfish and slender sole were often seen swimming over or resting near the carcass, but showed little direct interest. *Pandalus platyceros* picked constantly at the remains, leaving small marks in the tissue. In general the skin of the carcasses remained intact as the fauna removed the internal tissues and organs. Over this time, Carcass 3 remained unchanged, with silt depositing on the body and a few *M. quadrispina* around, but unable to pierce the skin.

**Figure 7:**
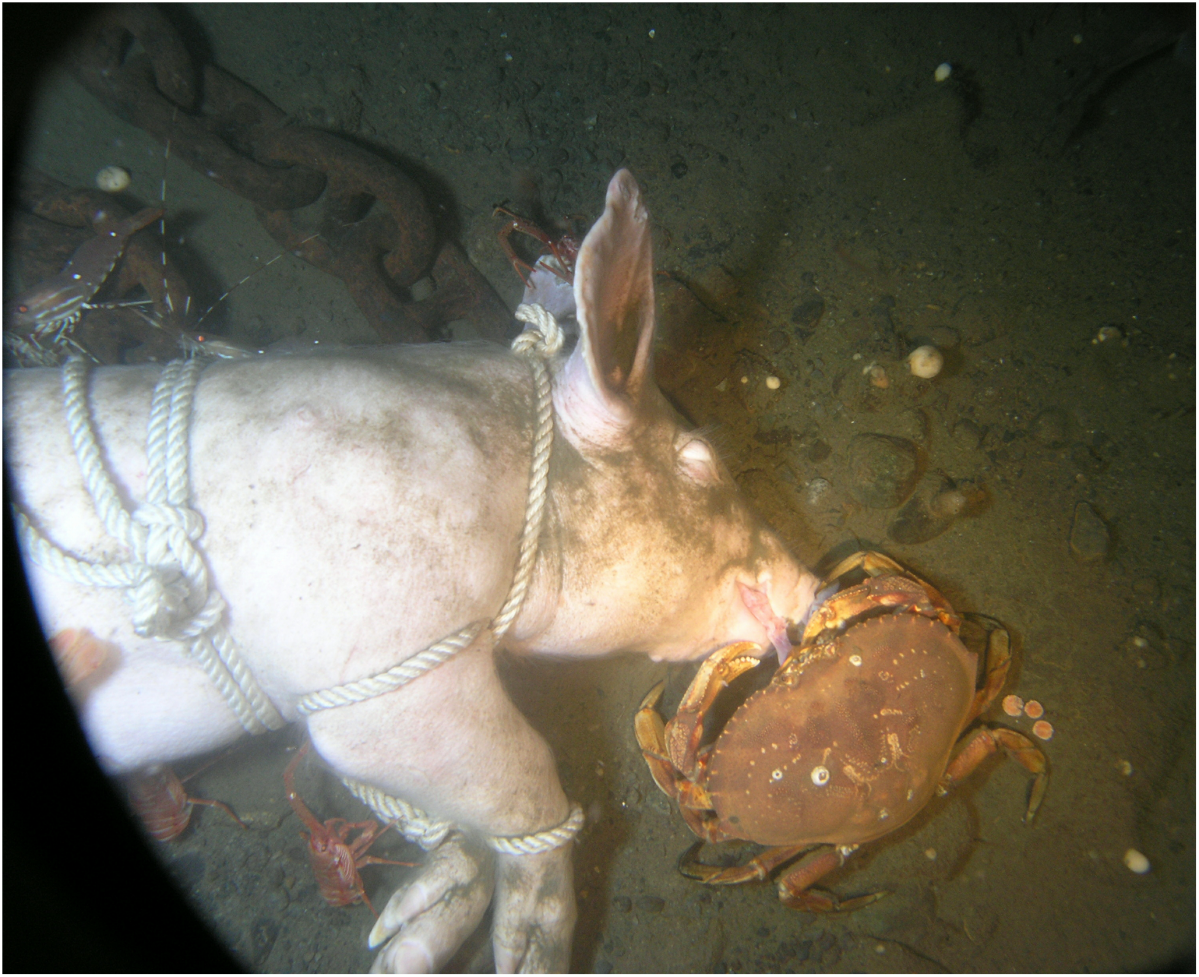
*Metacarcinus magister* Dana (Dungeness crab) pulling tongue from Carcass 2, Day 4. Note also *Munida quadrispina* Benedict (squat lobster) in lower left and on ear, and *Pandalus platyceros* Brandt (three spot shrimp) in upper left of picture (Ocean Network Canada’s VENUS observatory).

**Figure 8:**
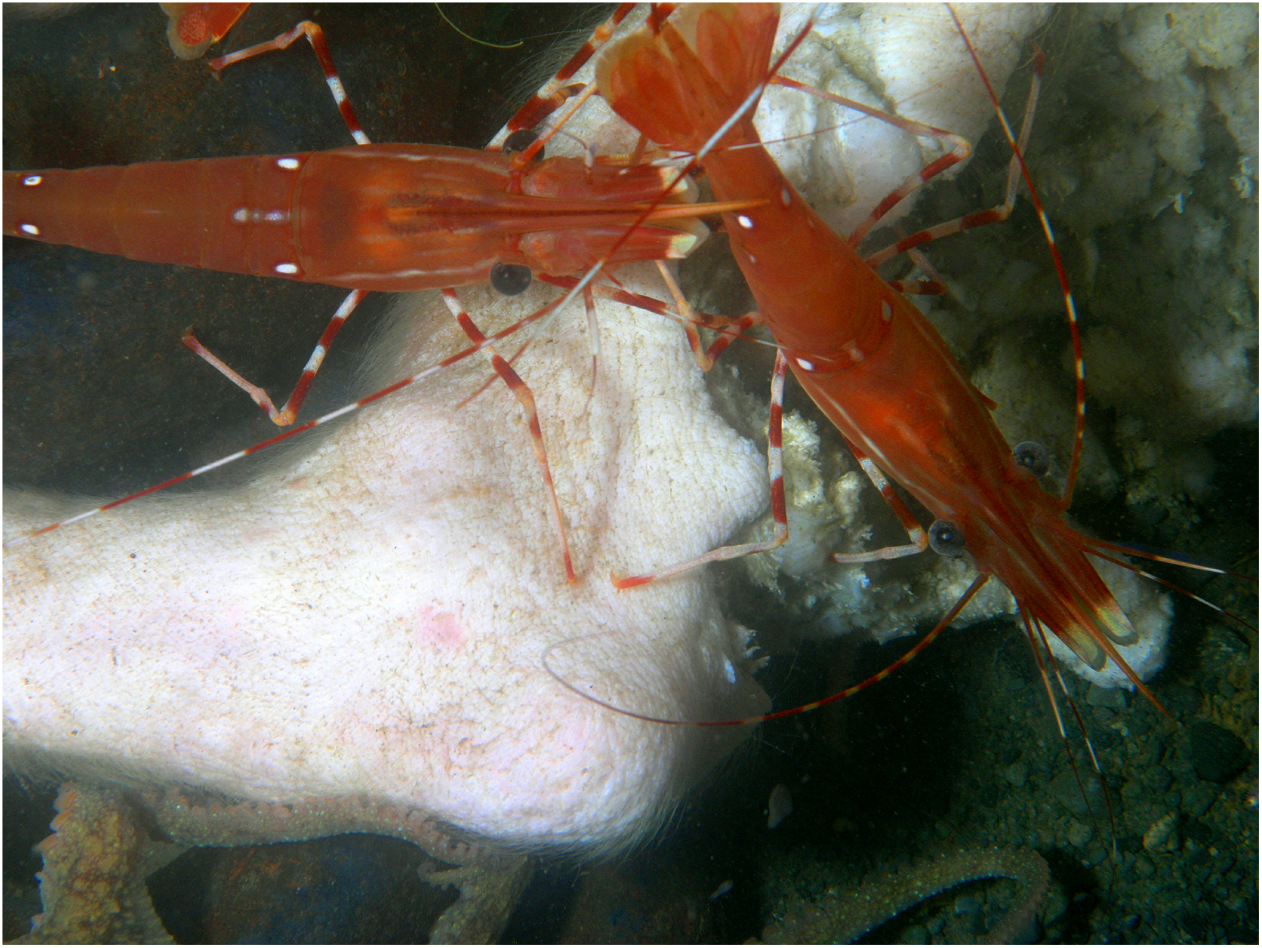
*Pandalus platyceros* Brandt (three spot shrimp) picking at the damaged area of left rear leg of Carcass 1 on Day 6. *Octopus rubescens* Berry (ruby octopus) at bottom of image (Ocean Network Canada’s VENUS observatory).

**Figure 9:**
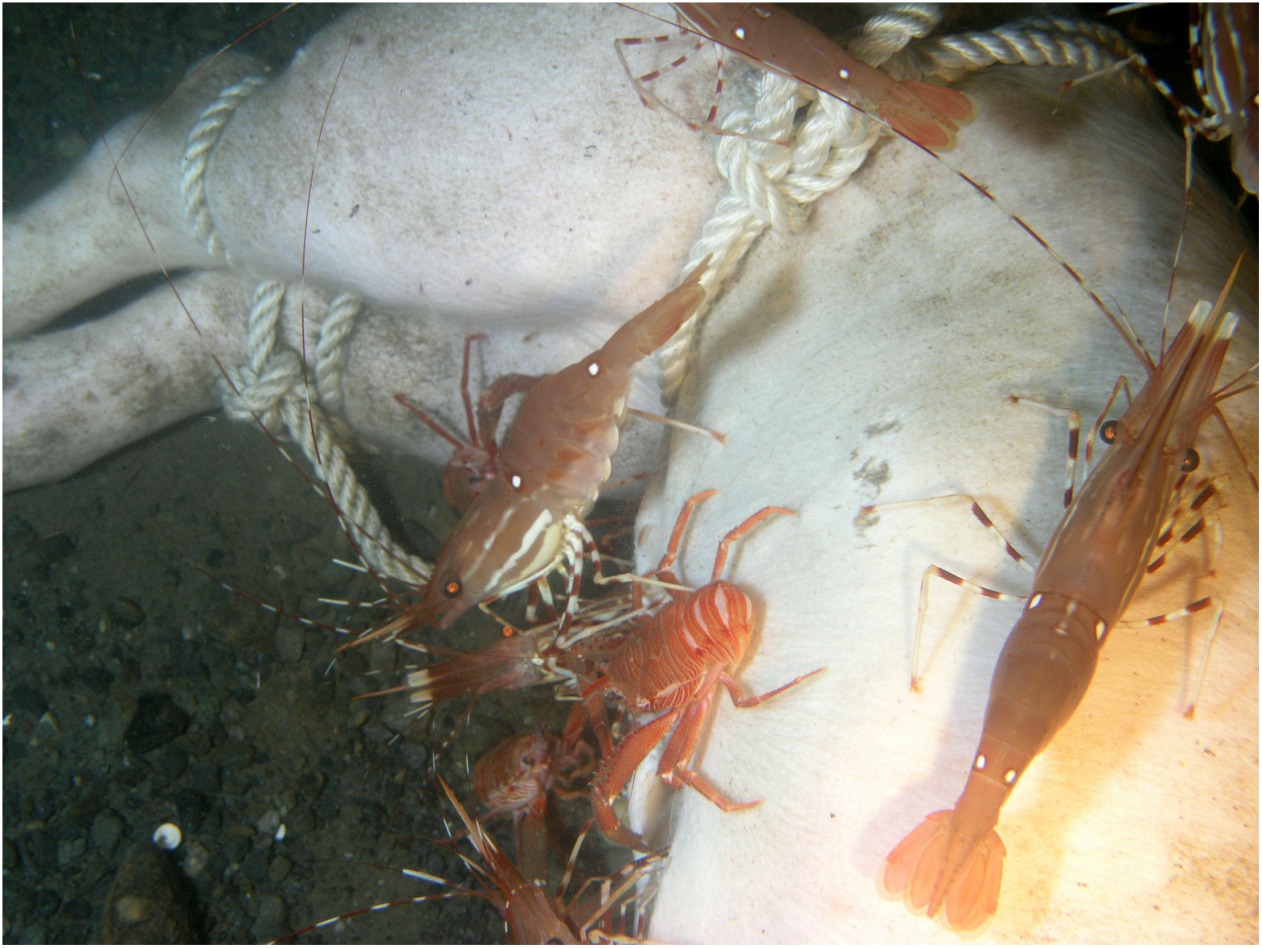
Artifacts in skin caused by *Metacarcinus magister* Dana (Dungeness crab). *Munida quadrispina* Benedict (squat lobster) and *Pandalus platyceros* Brandt (three spot shrimp) feeding at abdominal area when larger crabs not present and also feeding at claw marks (Ocean Network Canada’s VENUS observatory).

The entire abdominal area of Carcass 2 was opened by Day 3 and coils of intestine and organs were visible, with lengths being pulled out by the larger crabs. The crabs sometimes fed at the facial area and on Day 4 *M. magister* was seen pulling the tongue out of the mouth and consuming it (Figure 7). The artifacts created in the skin by *M. magister* were picked at and enlarged by *M. quadrispina* (Figure 8) and *P. platyceros* (Figure 9). In Carcass 1, as the scavenging had begun at the wound area rather than the abdomen, the abdominal cavity did not appear to be breached until Day 5 (Figure 1C), by which time much of the abdominal organs appeared to have been removed through the abdominal breach as this area appeared concave, and feeding had extended to the anus, between the back legs and the head, although the main site of activity was still the abdomen. Carcass 1 was briefly visited by several other species including a small *Pycnopodia helianthoides* Brandt (Sunflower sea star) and *Octopus rubescens* Berry (Ruby Octopus) which were attracted to the wound area. Crabs were seen to be rocking the carcass almost over and succeeded in moving it a further 15 cm. At some points up to six *M. magister* were present on the body with large numbers of *M. quadrispina* and *P. platyceros* present. Carcass 3 continued to exhibit little activity with only a few *M. quadrispina* present, but not damaging the skin. From Days 3–7, dissolved oxygen levels were 1–1.2 mL/L at Carcass 1, 0.7–0.8 at Carcass 2 but were at only 0.4 mL/L at Carcass 3.

By Day 8 the lower part of the spinal column of Carcass 1 was entirely exposed (Figure 1D) with bone and cartilage visible, and the lowest ribs exposed. *Metacarcinus magister* was seen to reach into the cavity and pull material out, as well as enter the abdominal cavity and reach under the carcass and their activities regularly lifted and moved the entire carcass. Carcass 2 at this time was still fully intact, with the main tissue removal from the abdomen although clear grazing marks were seen in the face and legs. *Metacarcinus magister* dominated at the abdominal area and was often seen grabbing at *M. quadrispina* (Video S6) and although *M. quadrispina* did seem to attempt to avoid the larger crabs, the resource was rich enough that they would return to the carcass despite the presence of *M. magister* again and again. Carcass 3 still exhibited little activity with a few *M. quadrispina* present and one or two picking at the skin, but no damage was visible.

By Day 11, Carcass 1 had been moved repeatedly by animal activity and the majority of the rear end of the carcass was completely removed with the back legs mostly skeletonized (Video S7). The front half of the body remained largely intact but the internal organs were removed. It was evident that the loss of the lower part of the carcass meant that the weights, all linked together, were no longer holding the carcass *in situ* as it was being pulled out of the ropes by the larger crabs and by Day 13, Carcass 1 had been pulled free of the weights, and was gradually pulled away from the camera. At this time, Carcass 2 was still intact but the tissue around the abdominal area had been grazed to expose adipose tissue and further extend the opening and by Day 12, the ends of the lower ribs were exposed (Figure 2C). On the rest of the carcass skin, with hairs visible, was still present. *Metacarcinus magister* opened up the anal area and actively pulled out tissue (Video S8). From Day 10 at Carcass 2 and Day 12 on Carcass 1 as oxygen levels dropped (0.6 and 0.9 mL/L respectively), there were many days when *M. magister* and *P. platyceros* were absent or few in number, although they were still seen, sometimes in large numbers. By far the majority of fauna during these days were *M. quadrispina* which fed all over the carcasses, and would enter the body cavity to remove tissue, and open up areas in the tissue from inside, and also graze the face, rarely breaking into the tissue (Video S9) unless already opened by *Metacarcinus magister* (Video S10). At the same time, Carcass 3 still did not exhibit any damage although a few *M. quadrispina* picked at the skin. From Days 8–13, dissolved oxygen levels were fairly steady at 0.8–1 mL/L at Carcass 1, dropping for Carcass 2 to 0.5–0.7 mL/L and dropping further for Carcass 3 to 0.2–0.4 mL/L.

*Orchomenella obtusa* Sars (Family Lysianassidae), a small red amphipod, was seen in very small numbers for the first time on Day 13 on Carcass 1 (oxygen 0.8 mL/L) and Day 14 (oxygen 0.7 mL/L) on Carcass 2, present only on open tissue. Only small numbers of this amphipod were ever observed on Carcass 1, but on Carcass 2 by Day 17 almost all the exposed areas of tissue were suddenly completely covered by a thick layer of *O. obtusa* making the tissue appear pink (Figure 2D). They appeared only attracted to the open areas of tissue, with no skin and went inside the carcass to feed on the internal tissues, beneath the skin.

By Day 15 Carcass 1 was pulled further from the camera and tissue pulled back to show the level of skeletonization (Figure 1E) of the rear half of the carcass. By later the same day one of the hind legs was disarticulated and moved by *M. magister* activity, despite the fact that oxygen levels had dropped to 0.5 mL/L. The following day, the carcass was turned around 180° allowing a clear view of the head area. The head and front end of the carcass were externally intact, with only some grazing marks from *M. quadrispina* visible around the snout and orbits (Figure 1F). The remains of the carcass were removed from the range of the camera by Day 22, with only a disarticulated femur visible by Day 23. In the latter days, the carcass fauna was dominated by *M. quadrispina* with some *M. magister. Pandalus platyceros* were not observed after Day 11, when oxygen levels dropped below 0.9 mL/L.

On Day 18, the rear half of Carcass 2 from mid spinal area was removed entirely. Although the tissue removal was not observed it is believed to have been caused by *H. griseus* the sixgill shark, as before. However, this time, due to the different weighting system, the front half of the carcass remained in camera range for the duration of the study. The rear half was never recovered. Large numbers of *O. obtusa* were seen on the exposed areas of tissue and fed on the internal soft tissues from the inside of the carcass, hollowing it out from inside out, so that by Day 21 the carcass began to appear as if it was just skeletal elements covered by skin, as the skin had a loose, wrinkled appearance, such as that of a loose shirt (Figure 2E). Very large numbers of *M. quadrispina* fed on the remains with only occasional visits by *M. magister* and *P. platyceros*, with oxygen levels down to 0.4 mL/L. The small crabs ripped and pulled at the skin and by Day 23 began to pull it over the top of the head, much like a shirt (Figure 2F). By Day 25 almost all the skin had been pulled off, revealing cleanly skeletonized yet mostly articulated bones and cartilage. Once the internal tissue had been removed, *O. obtusa* were no longer seen. The last pieces of soft tissue to be consumed were the ears. Once the soft tissue was removed, *M. quadrispina* still remained on the carcass, feeding on the cartilage and so disarticulating and moving the remaining skeleton. By Day 31 the ribs were disarticulated and the majority of cartilage had been consumed by Day 38, after which very few *M. quadrispina* or other fauna were observed.

**Figure 10:**
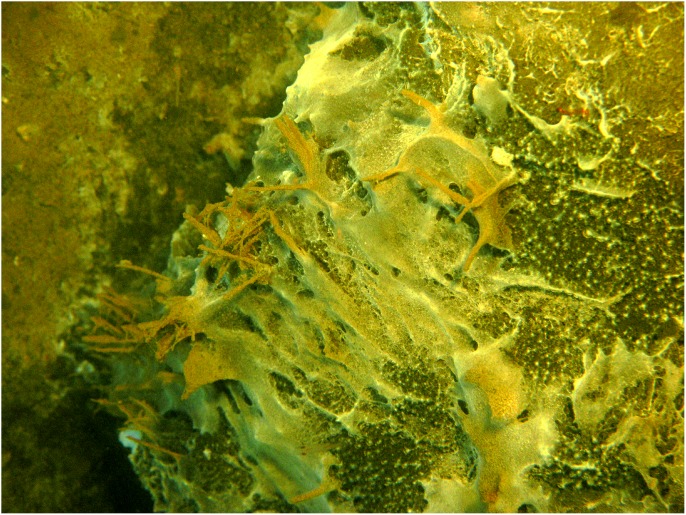
Sulphurous bacterial mat formed on carcass (Ocean Network Canada’s VENUS observatory).

Carcass 3, in contrast to both Carcass 1 and 2, remained only attractive to a very low number of *M. quadrispina*, and no damage was noted until Day 22 when a few shallow grazing marks could be seen on the nipples and inside of the rear left leg (Table 3). By Day 27 more small grazed areas could be seen in the inguinal and lower abdominal area but the damage was only skin deep and did not penetrate into the abdominal cavity (Figure 3C). One or two *M. quadrispina* were sometimes observed in the area and by Day 31 a thick filamentous sulphur bacterial mat was forming over the carcass, which continued to grow and thicken over the following weeks (Figure 3D). Sometimes *L. exilis* would rest on the carcass and slough away a patch of the bacterial mat, leaving exposed intact skin. Immature *M. quadrispina* were seen in the sand substrate by Day 46, but little activity was observed on the carcass and very little further damage was noted. Oxygen levels remained very low ranging from 0.2–0.4 mL/L. By Day 84, the bacterial mat was very thick (Figure 10) and little external change could be seen in the carcass. During this time, from Day 0-Day 88, dissolved oxygen levels were very low, ranging from 0.2–0.4 mL/L. On occasions, it did reach 0.5 and even 0.7 mL/L on one day (Day 44) at which levels some *M. magister* and *P. platyceros* were seen on Carcasses 1 and 2 but not on Carcass 3. However, by late December (Day 92), oxygen levels began to rise reaching 0.8 and eventually 1.5–2.2 mL/L (Figure 5) and very large numbers of a variety of species of fish were suddenly seen swimming over the carcass (Figure 3E), almost obscuring it at times. The very large numbers may have been an artifact of the light, but clearly large numbers of fish were in the vicinity as the lights had not attracted any vertebrate activity in the preceding four months.

Interestingly, despite the dramatic appearance of large numbers of vertebrates at Carcass 3, it was several days later (Day 98) before arthropods were seen on the carcass, when a few *M. quadrispina* were observed, together with the first appearance of one or two *P. platyceros* but by Day 106, large numbers of *P. platyceros* and several *M. magister* had joined the *M. quadrispina* and were actively feeding on the carcass breaking into the tissue. The majority of the shoulder and rump area as well as parts of legs and the central abdominal area were opened up and tissue exposed by the larger arthropods. Very large numbers of *Pandalus platyceros* fed on the carcass from this point on, together with *M. magister* although very few *M. quadrispina* were observed. No amphipods were observed.

Over the subsequent 30 days, the carcass was rapidly skeletonized, with some disarticulation, primarily by the constant feeding activity of the shrimp and crabs (Figure 3F). Complete skeletonization was not observed as the experiment was terminated at Day 135.

Throughout the observation of Carcasses 1 and 2 the fauna was dominated by three species, *M. quadrispina*, *P. platyceros* and *M. magister.* These species were primarily responsible for the removal of the soft tissue as well as the cartilage, and for disarticulating and moving the carcasses. All three major crustaceans remained at the carcass over the 24 h cycle and did not show any diurnal pattern. They fed continuously, removing muscle and organ tissue, then cartilage. Artifacts specific to each of the major crustaceans’ feeding patterns were observed. A fourth species, *O. obtusa,* arrived later and had a major impact on the soft tissue removal of Carcass 2. Decompositional stages and signs usually observed in bodies in water, such as bloat, putrefaction, active and advanced decay and skin slippage, were not observed in any of the carcasses. Tissue loss was entirely due to scavenger feeding, although this was greatly delayed in Carcass 3. No parts of Carcass 1 were recovered, despite extensive searches by ROPOS in the area three months later, but some skeletal elements from Carcasses 2 and 3 were recovered for future studies.

### Oceanic Physical and Chemical Measurements

Dissolved oxygen, temperature, salinity, density, conductivity and pressure were measured for all three deployments.

**Figure 11:**
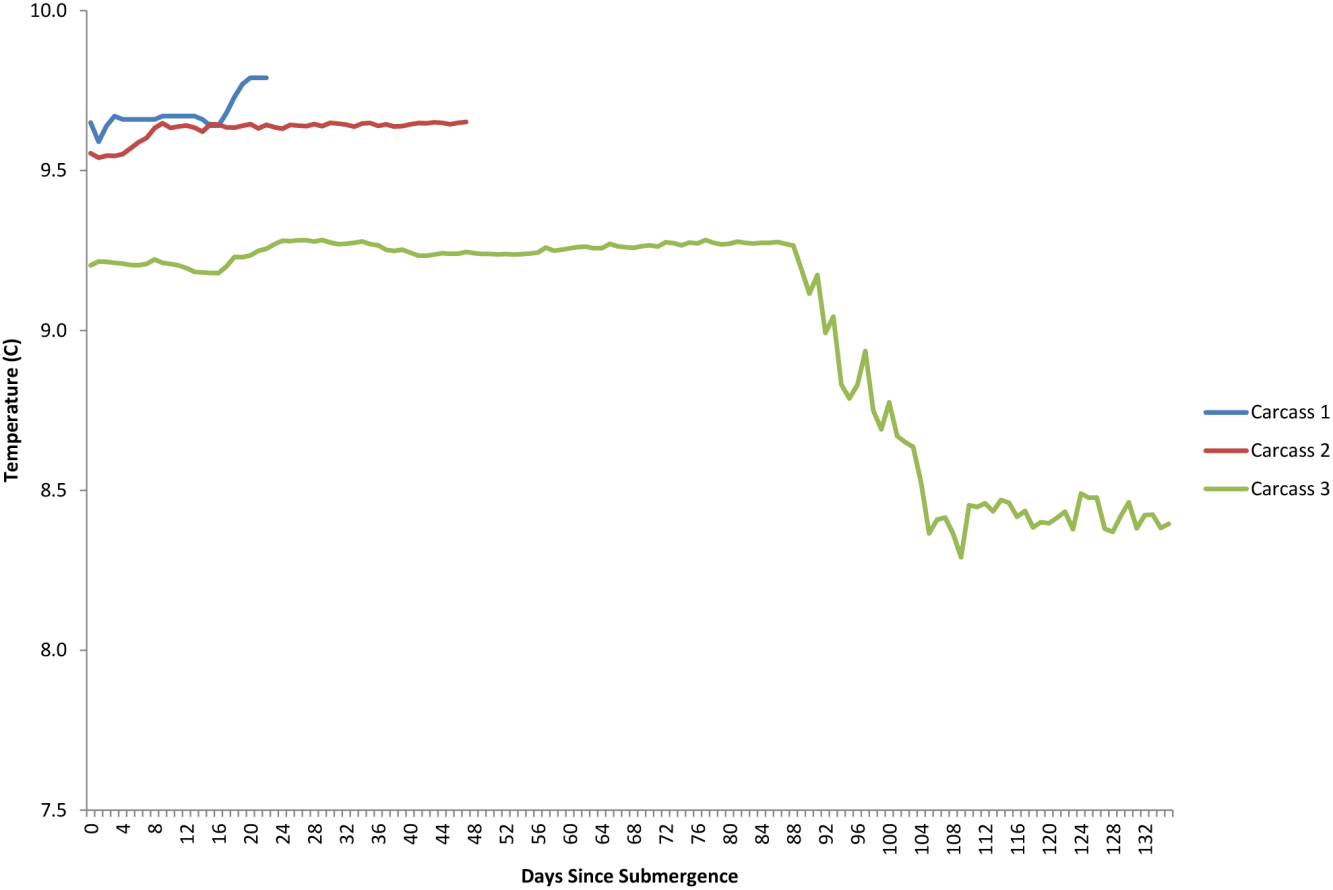
Temperature (°C) for the duration of study for each carcass (Ocean Network Canada’s VENUS observatory).

**Figure 12:**
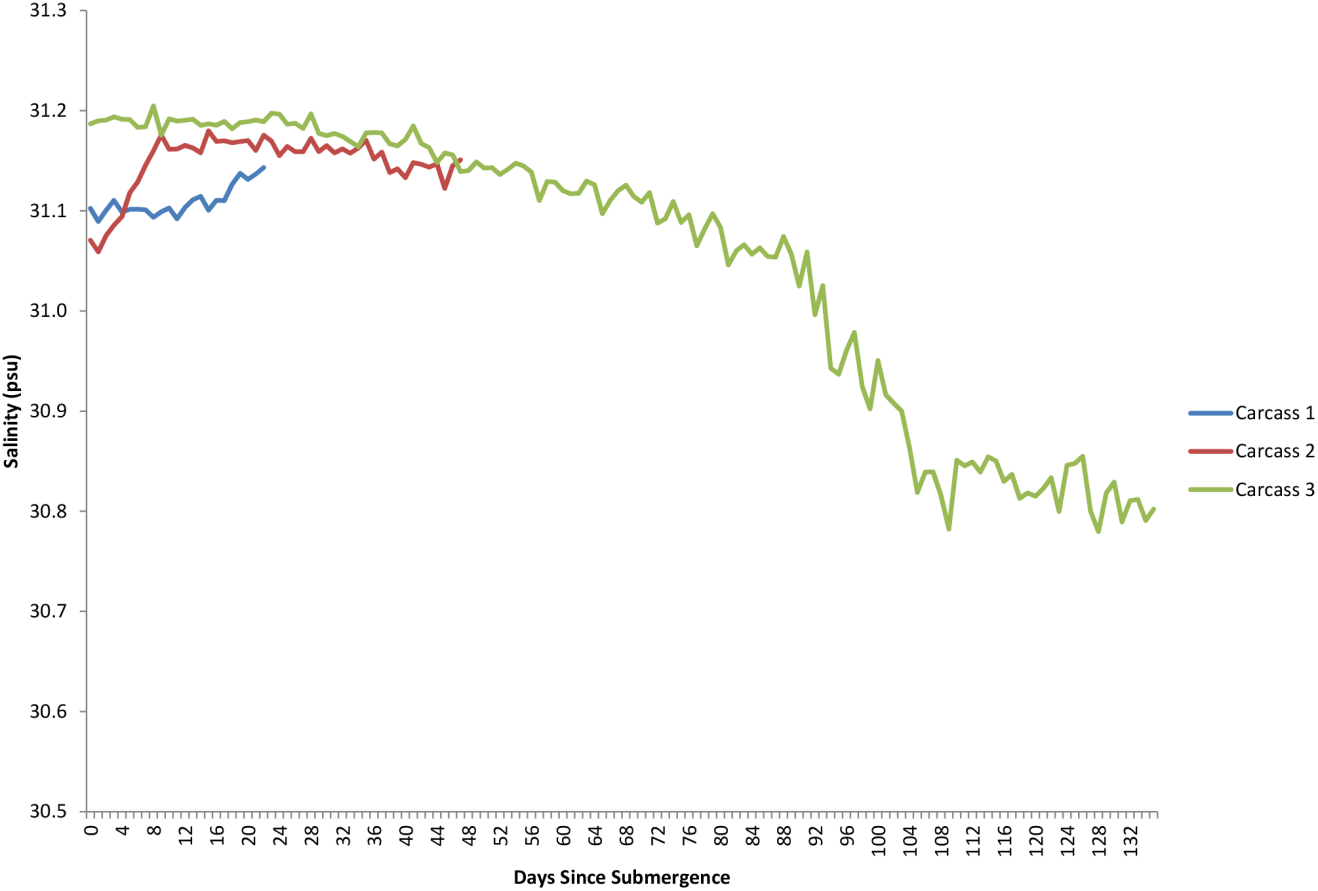
Salinity (psu) for the duration of study for each carcass (Ocean Network Canada’s VENUS observatory).

**Figure 13:**
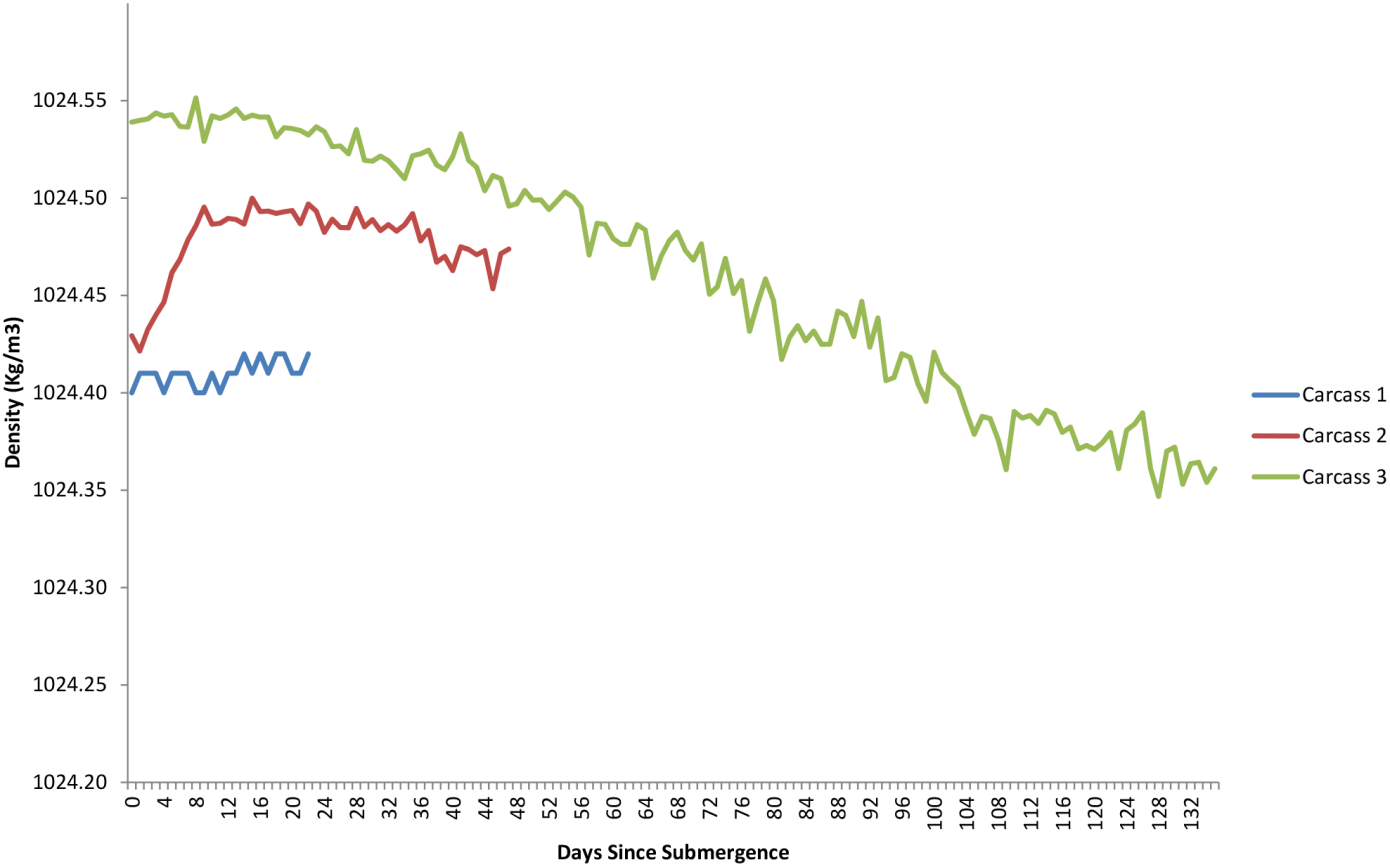
Density (Kg/m^3^) for the duration of study for each carcass. (Ocean Network Canada’s VENUS observatory).

**Figure 14:**
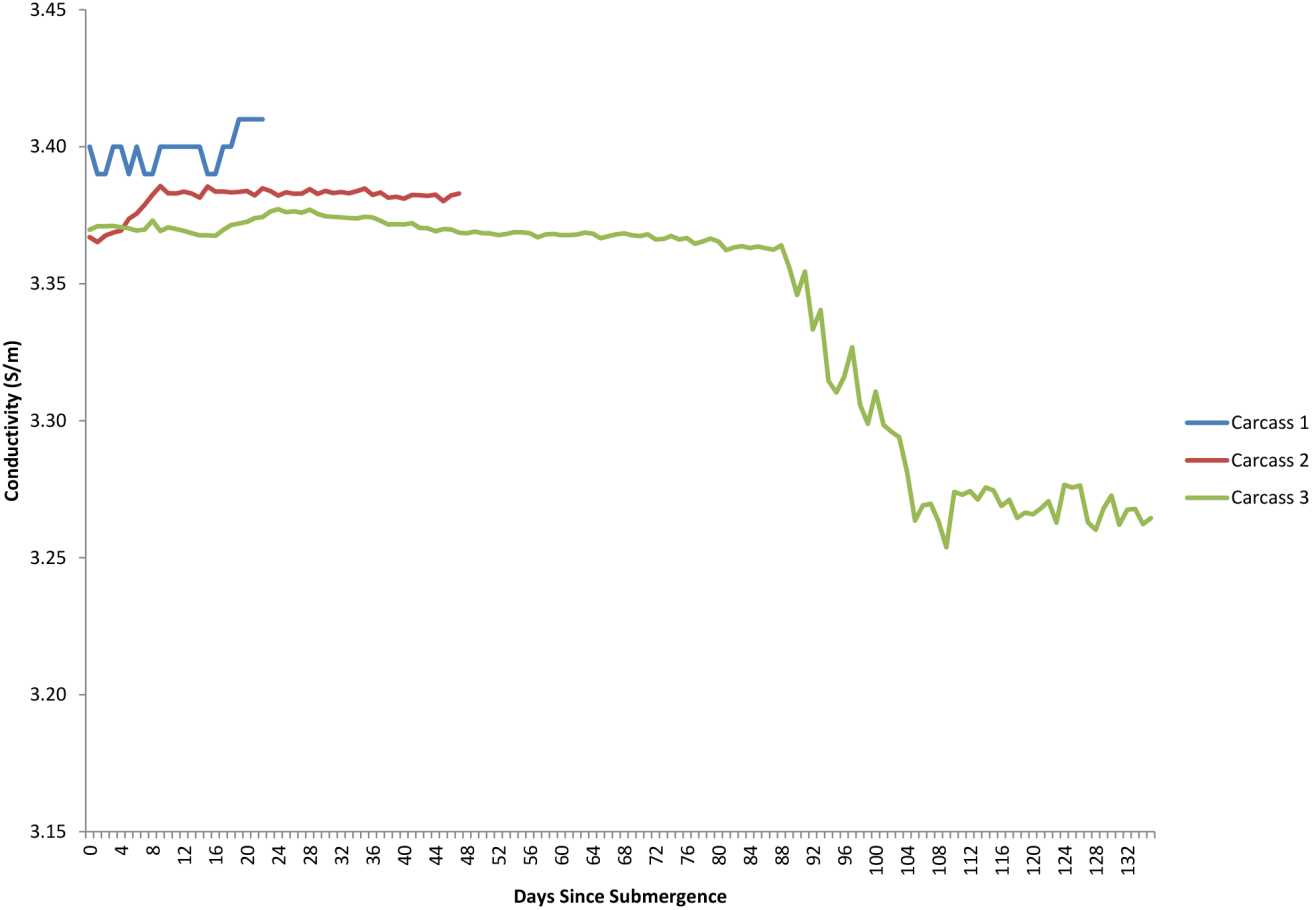
Conductivity (S/m) for the duration of study for each carcass. (Ocean Network Canada’s VENUS observatory).

**Figure 15:**
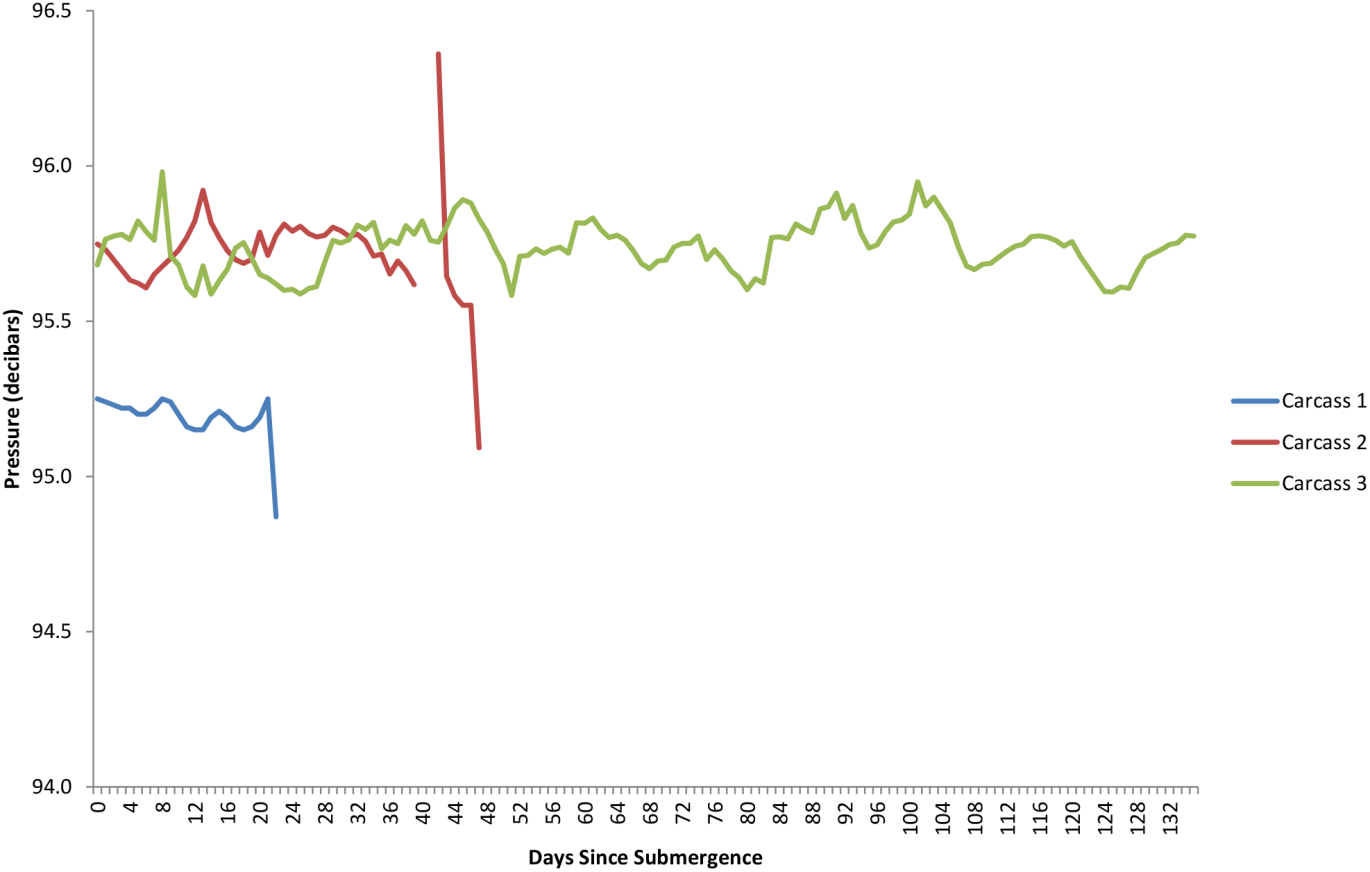
Pressure (decibar) for the duration of study for each carcass (Ocean Network Canada’s VENUS observatory).

The dissolved oxygen levels for the duration of each study are shown in Figure 5. The first two deployments occurred when dissolved oxygen levels were generally low, at or around 0.9–1.4 ml/L and these levels dropped over the period of study. The third deployment occurred when dissolved oxygen levels were markedly lower at 0.5 mL/L and dropped lower before increasing between 92 and 108 days post submergence. Temperature had an inverse relationship with oxygen levels, dropping as oxygen increased due to deep water renewal, however, despite fluctuations, it remained within 1–2 °C, ranging from 8.4–9.8°C (Figure 11). Salinity, density, conductivity and pressure also decreased inversely as oxygen increased (Figures 12, 13, 14, 15).

## Discussion

The scavenging progression of Carcasses 1 and 2 were very similar, with the immediate attraction of *M. quadrispina, M. magister* and *P. platyceros*, which proceeded to rapidly scavenge and skeletonize the carcasses. *Orchomenella obtusa* was also present on both carcasses although it was present in much larger numbers and had much greater impact on Carcass 2. The third carcass deployment, however, was quite different, with only *M. quadrispina* attracted at the beginning, followed by a long period with no arthropod activity, then a sudden upsurge of both invertebrate and vertebrate activity. The invertebrate activity and consequent scavenging of all three carcasses appeared to be a direct reflection of the dissolved oxygen levels in the water.

### Impact of Abiotic Parameters

The Saanich Inlet is naturally a low oxygen or hypoxic basin with seasonal anoxia. It is a narrow, deep fjord with a much shallower sill at its mouth, which prevents oxygenation of water in the deep basin for much of the year. Oxygen is increased in the spring and fall when cold, well oxygenated, dense water enters the basin over the sill from Haro Strait, displacing the deoxygenated water and affecting oxygen levels, temperature, conductivity, pressure, density and salinity [39], [40]. During much of the year, therefore, the deep water is hypoxic to anoxic. Dissolved oxygen levels below 2 mL/L are considered hypoxic and conditions become very stressful for most animals below 1 mL/L [41]. All the carcasses were deployed in the late summer/early fall period but the deep water renewal, although seasonal, can vary slightly temporally. Thus, the first two carcasses were deployed when the dissolved oxygen levels were hypoxic but still clearly acceptable for considerable invertebrate activity. However, the third carcass was deployed when the oxygen levels were very low and this greatly affected faunal activity.

In the very low oxygen conditions of the third deployment, only a few *M. quadrispina* were attracted and, although they grazed the skin, they were unable to break through to reach the organs or internal tissues. Clearly the larger crabs were needed to open the carcass for the smaller, more gracile crustaceans. In their absence, *M. quadrispina* was only capable of some light grazing on the surface, until the deep water renewal increased oxygen levels to allow the presence of the larger crabs and shrimp. Therefore, the dramatic difference between the first two deployments and the third appears to be directly driven by oxygen levels, primarily at time of deployment. Had the larger crabs been able to open the carcass first, it is likely that more *M. quadrispina* would have remained at the third carcass as oxygen levels, although low for most crustaceans, was perfectly acceptable for *M. quadrispina* as they are found in large numbers in oxygen levels as low as 0.1 mL/L [40], hence more tissue removal might have occurred. Human skin is considered to be similar to pig skin physically and physiologically [42], [43] so could be expected to react similarly to such scavenging.

During the period of low oxygen, a thick bacterial mat formed over Carcass 3 alone, occasionally being dislodged by *L. exilis* the slender sole, which is extremely tolerant of very low oxygen levels [44]. This pleuronectid flat fish feeds primarily on pelagic crustaceans and is important in bioturbation of the ocean floor, disturbing mat formation and aerating sediments [44]. When oxygen was extremely low, this was the only vertebrate observed. The bacterial mats are created by filamentous sulphide-oxidizing bacteria which form a biofilm close to a site with both hydrogen sulphide and low levels of dissolved oxygen. Most are micro-aerophiles requiring low levels of oxygen to metabolize but are unable to survive in anything but extreme hypoxia [31], [45]. Such mats were not observed on the first two carcasses, probably because, although the waters were still hypoxic, the oxygen levels would have been too high for survival. Such sulphur mats are common during extreme hypoxia in Saanich Inlet [44] and have frequently been observed on whale falls [46].

Even though oxygen levels were higher during the first two Carcass deployments, conditions were still hypoxic for the duration of both studies, with levels below 1 mL/L from Day 9 onwards for Carcass 1 and from deployment for Carcass 2. However, the presence of such a rich nutrient source clearly attracted large numbers of crustaceans despite the low oxygen. Even when the oxygen continued to drop even lower, most of the crustaceans remained, despite the increasingly stressful conditions. *Metacarcinus magister* has previously been shown to prefer higher oxygen conditions, whether fed or unfed, and laboratory experiments showed that when specimens did enter hypoxic conditions to feed, they carried the food to higher oxygen conditions to consume [47]. Field experiments also showed they preferred to remain in higher oxygenated waters to digest. Fed and unfed crabs were released with ultrasonic telemetry tags into Bamfield Inlet in Barkley Sound, British Columbia (off the west coast of Vancouver Island) and tracked for 48 h. Unfed crabs were found to move over 1300 m in 6 hours, whereas fed crabs moved directly to higher oxygen areas and remained mostly immobile, suggesting they selectively chose a higher oxygen, less stressful environment in which to digest [47]. This is very different from that which was observed in the present experiments where crabs were attracted to the first two carcasses immediately and appeared to remain with the carcasses almost continuously for several days at a time. Barnacle patterns on the carapaces of the crabs appeared to be highly individualizing, allowing tracking of individual crabs. The crabs did not remove tissue to take to a higher oxygen area to eat or to digest, but appeared to remain with the carcass for long periods of time, feeding directly on the carcass, and sometimes on the other crustaceans. The carcasses were only monitored several times a day, so it is possible the crabs could have left and returned, but their continued observation suggested they did remain for long periods of time. Although perhaps preferring higher oxygenated waters, the presence of such a rich nutrient resource clearly outweighed the costs of the stressful conditions. Decapod crustaceans such as *M. magister* have developed several mechanisms to cope with hypoxic conditions. In low levels of dissolved oxygen decapods can increase haemolymph flow over the branchia [48], but this is only effective down to a certain level of dissolved oxygen where mechanisms such as brachycardia [47] and redirection of haemolymph flow rates to limbs or tissues requiring greater oxygen levels may also come into play [49]. The fact that the crabs remained feeding and digesting at the carcass for long periods of time, despite hypoxia, may be dependent on the quality of the resource. Bernatis et al. [47] used fish muscle in their feeding experiments whereas, in the present study, the crabs had access to a variety of tissue types, including organ and muscle.

When oxygen levels dropped very low, *M. magister* and *P. platyceros* were excluded and only *M. quadrispina* remained at the carcasses. This is consistent with previous studies in Saanich Inlet where *M. quadrispina* was found in areas with oxygen levels as low as 0.1 mL/L, although only the largest specimens were able to tolerate such low levels [40]. *M. quadrispina* living in such low oxygen conditions were sedentary and showed no aggression or territoriality despite occurring in very large numbers and in very close proximity, whereas specimens in more oxygenated waters were more aggressive and territorial and avoided contacting each other [40]. In the present study, *M. quadrispina* were often seen simply resting on or near the carcass when oxygen levels were very low, although they were also observed actively feeding and moving towards the carcasses. *Munida quadrispina* naturally feeds on live zooplankton but are obviously facultative scavengers when opportunity presents.

In general, most crustaceans are not tolerant of severe hypoxia, but *M. quadrispina* is an exception and has been shown to tolerate very low oxygen levels quite well [40], [50]. Larger *M. quadrispina* have a slower respiration rate and larger gill weight than smaller specimens in the same area as well as similar sized specimens from well oxygenated waters, allowing them to tolerate extreme hypoxia [51]. This is advantageous as such low oxygen, deep regions are also rich in nutrients [40] and lacking in predators [51]. This was confirmed in this study in that larger predators, such as *M. magister,* were excluded in extreme hypoxia.

Temperature decreased inversely with increased dissolved oxygen levels. In surface waters this occurs simply because oxygen solubility in water increases as temperature decreases, but in these deeper waters, the correlation is due to the seasonal deep water renewals during which there is a sudden massive influx of cold, dense, oxygenated water over the shallow sill at the mouth of Saanich Inlet, and into the basin, displacing the deoxygenated water with oxygen rich, cold water. This also impacts the salinity of the water, as well as the pressure and conductivity. However, it appears that oxygen levels alone drive the faunal colonization in this situation, as it is only oxygen that varied so dramatically.

Temperatures for all three deployments only varied by approximately 0.6°C over the first few weeks and even when temperature dropped during the deep water renewal of the third deployment, it still varied by only approximately 1°C. Therefore temperatures remained relatively similar at around 9.2–9.8°C for most of the time, with a drop to 8.4 °C during deep water renewal. As these temperatures are considered relatively warm, and were fairly consistent for most of the time, it is unlikely that temperature was a driving condition, and should have been relatively optimum for faunal colonization. *Pandalus platyceros*, for instance, prefers a temperature range of 8–11°C [52].

Salinity has been shown to have an impact on metabolic rates of crustaceans in many studies [53]–[56] and differences in animal decomposition and fauna colonization between fresh [57] and salt [28] water have been documented but the direct effects of salinity levels on decomposition have not been studied. Most species of crustaceans are osmoconformers, restricted to a relatively narrow specific salinity range. For instance, *P. platyceros* requires a salinity range of 26–31 psu whereas other *Pandalus* sp. can tolerate much wider ranges [52]. However, the differences in salinity over the three studies were very small and salinity remained around 31.1–31.2 psu so it is unlikely that any differences observed were due to salinity changes.

### Faunal Scavenging

In the first two deployments (Carcass 1 and 2), three major crustaceans, *M. magister*, *P. platyceros* and *M. quadrispina* were immediately attracted to the remains, with only the latter being attracted to the third carcass. All three fed continuously at the carcass, with no specific diurnal patterns as feeding continued through the day and night. *Munida quadrispina* are endemic in the Saanich Inlet and would have been in the vicinity of the carcasses when they were deployed, often in very large numbers [40]. However, they did not just feed opportunistically but were specifically attracted to the carcasses. Large numbers of them could be seen moving towards the carcass and their density was much greater at the carcass than in the general surrounding area, with densities of *M. quadrispina* up to 1.6/dm^2^ at the carcass site *versus* only 0.7/dm^2^ in other regions nearby [58]. They were attracted in very large numbers to the intact Carcasses 1 and 2 despite the fact that it was later shown that they could not break the skin without the presence of the larger crustaceans. *Metacarcinus magister* and *P. platyceros* are also endemic in the Inlet but are not normally found in the research area, so were also directly attracted to the carcasses. The research area substrate of fine silt over cobble is not favoured habitat for *P. platyceros* as they are more usually associated with rocky terrain [52] *s* being found in greatest numbers in slightly shallower waters, at 70–85 metres [59].

Chemoreception and mechano-reception to hydro-acoustic stimuli are important in attracting fauna to a food source in the marine environment. Mechano-reception has been shown to be important for *Pandalus* sp. in detecting a carcass with the shrimp either detecting the actual sound of the fall of the carcass to the ocean floor or responding to acoustic stimuli generated by conspecifics on the carcass [60]. However, in the present studies, much greater hydro-acoustic stimuli would have been generated by the actions of the submersible, ROPOS, when it placed the carcasses, which may have been the primary initial attractant, together perhaps with the lights on the submersible.

A fourth crustacean, the sea louse, *O. obtusa*, was seen in small numbers on the first carcass and in large numbers on the second carcass, although it did not appear until Days 13 and 14 respectively. Anecdotal information from rescue divers had suggested that the carcasses would be immediately attractive to large numbers of these amphipods and that they could completely obscure a cadaver and skeletonize exposed tissues in a matter of hours after death [13]. *Orchomenella* sp. have been reported to eat large pieces of seal meat in less than 24 h and were collected in vast numbers in the McMurdo Sound area of the Ross Sea (Antarctica) [61] cited by [12]. They are known to be common in the Saanich Inlet, being found from 80–210 metres and were found in very large numbers feeding on dead and dying prawns killed in a mass anoxic event [59]. Therefore, it was surprising that they did not colonize more rapidly and in greater numbers. In the second deployment, once they colonized, they did remove a large amount of biomass, entering *via* the already opened areas of the carcass and feeding from the inside out, leaving just skin lying on bone.

Aside from the four major crustaceans, very few species attended the carcasses. Occasional visits by *Chionectes tanneri* (the Tanner Crab), *Octopus rubescens* and *Pycnopodia helianthodes* were recorded, although in the latter case, the specimen was much smaller than those observed in shallow water experiments where animals much larger than the carcass completely enveloped it and were seen actively moving towards the carcasses [28],[30]. This low diversity is in contrast to earlier studies at 7.5 and 15.2 m where the carcasses attracted a rich diversity of fauna although the numbers in each taxon were very few. On many occasions, only one or two animals were observed [28], [30]. In the present studies, large numbers of each taxon were frequently present, but very little diversity was observed. This is not due to the unusual hypoxic environment as previous work in the Saanich Inlet shows that despite the conditions, the area supports a rich faunal diversity and abundance [33]. In contrast with terrestrial carcass faunal colonization, there was no evident successional pattern observed in these experiments, nor in studies in shallower waters [28], [30].

In studies in shallower waters, wounds and orifices were more attractive than the rest of the carcass for the first few hours after placement then the entire carcass appeared equally attractive [28], [30]. In the present studies, although the first arrivals did explore the orifices, these did not appear to be significantly more attractive than other areas of the carcass. However, when a large wound was created by a shark bite in Carcass 1, all the crustaceans were immediately attracted to this site and remained almost exclusively at this site for the duration. Some feeding damage was noted at the snout area due to *M. quadrispina* feeding, but this was probably in an effort to avoid predation by the large *M. magister* feeding at the open wound although, at most times, *M. quadrispina* and *P. platyceros* risked predation and fed beside *M. magister* due to the high quality of the resource.

Carcass 2 was not damaged by a shark until much later, so the primary feeding site was the abdominal area, with *M. magister* opening up the abdomen within 24 h of deployment. Once this area was opened, this became the major feeding site, with all three crustaceans feeding together and extending this area. Some feeding also occurred at the head and anal area, but the main site was the abdominal opening, extending into the inguinal area and up to the front legs. The carcass was bisected by a shark on Day 18 but, by this time, the carcass was heavily scavenged so the extra damage did not have a major impact on feeding patterns.

Only *M. quadrispina* was originally attracted to Carcass 3, also focusing on the abdominal areas, although their chelicerae were not strong enough to pierce the skin. Hence in these studies, when a wound was present, it was the first and continuous site of feeding, but when no damage was present, the crustaceans’ primary site of feeding was the abdominal area, rather than orifices.

The first two carcasses were *consumed* rather than decomposed, and the third carcass remained in stasis, with a thick bacterial mat forming over it, until oxygen levels increased and it too, was consumed. This is quite different from experiments conducted in shallower waters where, although the carcasses were scavenged, classic stages of decomposition, including bloat, active and advanced decay, were also observed [28], [30]. In previous shallow water experiments, the carcasses originally floated when placed, due to high adipose levels, then sank but refloated later due to bloat, caused by development of gases in the carcass from bacterial activity. Indeed, some of the carcasses retained gases in organs such as the stomach, which caused a false bloat and kept the carcass above the substrate for weeks. This impacted the fauna that scavenged the remains as those that remained floating were less accessible to animals that do not swim, while those that sank to the substrate (bottom) were much more rapidly scavenged [28], [30]. Although originally all carcasses were placed on a similar substrate, the bloat and false bloat had the effect of moving the carcass, which would then drop to rest randomly on sand or rock, and very different fauna colonized carcasses on each substrate [28], [30]. In the experiments presented here, the carcasses were placed directly on a consistently similar substrate and the lack of bloat meant they remained in direct contact with the substrate for the entire experiment.

### Forensic Significance

The present work has forensic significance for bodies recovered from the ocean, providing information on estimates of probable submersion interval, body movement, recovery expectations and identification of post mortem artifacts.

Although faunal succession was not observed, very distinct scavenging patterns were reported, and skeletonization was driven by faunal activity, which in turn was driven by dissolved oxygen levels. Although, these and previous studies show that it is unlikely that arthropod succession can be used to estimate a minimum submergence time, decompositional patterns related to substrate type, oxygen levels, effects of water activity and faunal species scavenging, may be helpful in indicating length of time in the water as well as habitat, substrate and geographical area, and what water body it may have first entered. Several past cases have indicated the value of understanding these parameters. Human remains were recovered after a submersion interval of over 30 years but the condition of the body suggested that the upper and lower halves had been exposed to very different environments, with the upper body showing considerable damage and the lower part of the body showing almost no damage [12]. The authors also noted that the upper body had clearly been in a well oxygenated environment as octopus eggs were found in the clothing whereas the condition of the lower part of the remains suggested they had rested in an anoxic environment, leading the authors to conclude that the body had been partially buried in silt [12]. In another case, a body was found a month after the person was last seen alive, in clam flats at the mouth of a river but shell fragments from marine animals and a spine of a sea urchin indicated that the decedent had not drowned in the flats but had entered the water in the ocean and had been swept into the flats [12].

Bodies lost in shallow water have been shown to first float, sink then bloat so could easily be transported from the deposition site by currents or tides [28], [30]. Therefore, in such cases, recovery efforts should be concentrated not just at the site last seen but also along tidal or current direction. However, human bodies which drop to depths below approximately 61 m (200 ft) do not bloat due to high pressure which reduces the gas volume as well as resulting in the gases becoming very soluble in tissues and water [13]. Therefore, it might be assumed that such remains would be found close to the site last seen. However, the results of these studies clearly show that animal activity can move a body rapidly. The part of Carcass 1 which was dragged from camera view was never recovered despite extensive searches by ROPOS in the area.

The results from these studies also provide realistic expectations for recovery efforts. It is clear that if a body goes into the ocean under conditions similar to those of Carcass 1 and 2 it will be scavenged and, if not weighted or tethered, will be moved from the original site rapidly. However, if time of year and condition more closely approximate the conditions of Carcass 3 then it is possible that remains may be found intact months after submergence. This is important information for recovery divers and also for managing expectations of family members.

This study also identified several important post-mortem artifacts caused by animal feeding. The largest and most obvious damage was believed to be caused by a six-gill shark, *H. griseus,* a deep water and wide ranging shark species [62]. Its diet consists of bony and cartilaginous fish although it has also been reported to occasionally consume invertebrates and marine mammals [63]. In the present studies, both bites occurred at night, between ∼1800 h and 0800 h local time which also fits with *H. griseus’* known feeding habits as it known to rest in deeper waters during the day then swim to shallow waters to feed at night [19]. The damage in this case could be identified as a shark bite due to the very clear tooth marks in the carcass (Figure 1B).

Much smaller artifacts were created by crustacean feeding. Crab claws, either picking at the tissue, or anchoring the crab while feeding, created damage that could be misinterpreted as sharp instrument trauma if not understood. As well, shrimp and *M. quadrispina* feeding created specific and identifiable artifacts. Similar damage from crabs has been observed in the Japan Sea [64] and crater-like defects have been observed on bone [12], [65].

It is extremely important that such artifacts are correctly interpreted, as misinterpretation has led to serious miscarriages of justice. For example, in Mississippi, Kennedy Brewer was sentenced to death for the rape and murder of a three year old child based on the marks on her skin alleged to be bite marks caused by the front teeth of the defendant. After years on death row Dr. John Wallace, a Board Certified Forensic Entomologist was called into the case by the Innocence Project and experimentally demonstrated that the alleged bite marks were caused by crayfish living in the stream from which the child’s body was recovered. DNA later identified her true killer [66].

The present studies have also shown that faunal activity alone can move a body, and disarticulate limbs quite rapidly. This is important as it is not uncommon for disarticulated human appendages to be recovered washed up on beaches, leading to media speculation of dismemberment and foul play. These studies show that such disarticulation can be a normal result of crustacean activity.

Other artifacts can be created by tidal activity and currents moving the body against abrasive substrates. For instance, bodies washed ashore often exhibit damage to hands, face and knees, which, if misinterpreted as pre or peri-mortem injury might suggest that the person had been in a fight prior to death [13]. In shallow water studies, various post mortem artifacts were reported, caused by the currents moving the carcasses against rocks and also by animal feeding, including surface skin damage as well as deep wounds that could be mistaken for an incision [28], [30]. Bodies that come into contact with boats may exhibit deep incised wounds due to propeller blades, and such remains must be carefully examined to ensure the post-mortem damage is not obscuring pre-mortem injuries. In a case in Florida, the badly decomposed remains of a man bore evidence of trauma from propeller blades, but careful examination indicated trauma to the ventral and dorsal regions of the body which were more consistent with blunt force trauma and the case was listed as a homicide [67].

### Conclusions

The experiments described here showed the impact of submergence and faunal scavenging on pig carcasses in a predominantly hypoxic environment at a depth of 100 m. The first two carcases progressed very similarly despite the removal of a large piece of Carcass 1 by a shark shortly after deployment. Both carcasses immediately attracted a large number of *M. quadrispina* which approached the carcasses from all angles in legions, clearly greatly attracted by the carcasses. *Munida quadrispina* was rapidly followed by *P. platyceros* and *M. magister* and all began to feed on the carcass. Feeding was concentrated at the damaged area on Carcass 1 allowing easy access to soft tissue and organs, but Carcass 2 was breached almost as fast by the large crabs ripping open the abdomen, so that tissue removal was similar for both carcasses. Although the three carcass deployments were meant to replicate each other, the timing of the final carcass deployment meant that this carcass was exposed to very different abiotic conditions which greatly impacted the faunal scavenging. Due to very low oxygen levels when Carcass 3 was deployed, the larger crustaceans were excluded, which showed that although *M. quadrispina* were attracted (albeit in low numbers) they are not strong enough to break the pig skin and require the presence of the larger crustaceans to give them access to soft tissue. It is probable that this would be the same in human tissue as pig and human skin are very similar. However, once the skin had been breached, these small crabs removed a great deal of tissue and were solely responsible for the removal of the last parts of soft tissue and cartilage and even skin on Carcass 2, disarticulating and moving the skeleton alone. Therefore, their role in carcass breakdown should not be underestimated, despite their inability to breech the carcass alone.

Although there was variation in all the abiotic parameters measured, it was only dissolved oxygen levels that had a dramatic impact on faunal scavenging and hence carcass breakdown. Oxygen levels were below 2 mL/L when Carcass 1 was deployed and dropped rapidly to 1 mL/L by Day 2 and were below 1 mL/L from deployment to completion for Carcass 2 but despite these stressful conditions, the large crustaceans rapidly fed on the remains, remaining at the carcasses despite the hypoxia, indicating that although such oxygen levels are considered very stressful normally, the high value of the resource outweighed the costs. As oxygen levels continued to drop however, the larger crustaceans could no longer tolerate the conditions and only *M. quadrispina* remained, as it is extremely well adapted to low oxygen conditions. When Carcass 3 was deployed during extreme hypoxia which excluded the larger crustaceans, the carcass remained intact for months and developed an extensive bacterial mat which was only destroyed when deep water renewal introduced cold, highly oxygenated waters, allowing access to the crustaceans again.

These studies have provided valuable information for underwater death investigations, describing conditions of bodies over time in hypoxic and anoxic environments. This provides data which will aid in estimating submersion interval, understanding the probable marine conditions to which a body has been exposed, explaining body condition such as disarticulation and body movement as well as illustrating a variety of faunal post mortem artifacts which, if not understood, could be misinterpreted as pre-mortem injury. They provide information to recovery divers and families as to the expectations of body conditions when in similar water conditions which will all assist in water recoveries.

Studies such as these are logistically complex to conduct due to the nature of the underwater environment, including risks to human divers, time and weather limitations as well as the requirement for vessels and divers. VENUS however provides a perfect vehicle for such studies as it only requires a small window of time with ideal conditions to deploy the carcass and then the experiment can be undertaken remotely in real time, with continuous data collection. Thus VENUS has provided a unique opportunity to investigate the fate of carrion in the ocean.

These experiments are ongoing, with studies being conducted in a variety of marine habitats and seasons (www.oceannetworks.ca).

## Supporting Information

Video S1 **Carcass 1, Day 0.** First view of carcass on seabed. *Munida quadrispina* Benedict (squat lobster) actively attracted (Ocean Network Canada’s VENUS observatory).
(MPG)

Video S2 **Carcass 1, Day 4.** *Munida quadrispina* Benedict (squat lobster), *Pandalus platyceros* Brandt (three spot shrimp) and *Metacarcinus magister* Dana (Dungeness crab) feeding on carcass (Ocean Network Canada’s VENUS observatory).
(WMV)

Video S3 **Carcass 1, Day 6.** *Munida quadrispina* Benedict (squat lobster) and *Pandalus platyceros* Brandt (three spot shrimp) feeding on carcass (Ocean Network Canada’s VENUS observatory).
(WMV)

Video S4 **Carcass 2, Day 2.** Several *Metacarcinus magister* Dana (Dungeness crab) opening and feeding in abdominal area (Ocean Network Canada’s VENUS observatory).
(MPG)

Video S5 **Carcass 1, Day 10.** *Metacarcinus magister* Dana (Dungeness crab) rocking entire carcass and lifting it from seabed (Ocean Network Canada’s VENUS observatory).
(MPG)

Video S6 **Carcass 2, Day 6.** *Metacarcinus magister* Dana (Dungeness crab) chasing a *Munida quadrispina* Benedict (squat lobster) (Ocean Network Canada’s VENUS observatory).
(MPG)

Video S7 **Carcass 1, Day 11.** *Metacarcinus magister* Dana (Dungeness crab) feeding on carcass (Ocean Network Canada’s VENUS observatory).
(MPG)

Video S8 **Carcass 2, Day 11.** *Metacarcinus magister* Dana (Dungeness crab) feeding at anus (Ocean Network Canada’s VENUS observatory).
(MPG)

Video S9 **Carcass 2, Day 12.** *Munida quadrispina* Benedict (squat lobster) feeding on face (Ocean Network Canada’s VENUS observatory).
(MPG)

Video S10 **Carcass 2, Day 16.** *Munida quadrispina* Benedict (squat lobster) feeding at abdominal area (Ocean Network Canada’s VENUS observatory).
(MPG)
